# A tool to automatically analyze electromagnetic tracking data from high dose rate brachytherapy of breast cancer patients

**DOI:** 10.1371/journal.pone.0183608

**Published:** 2017-09-21

**Authors:** Th. I. Götz, G. Lahmer, V. Strnad, Ch. Bert, B. Hensel, A. M. Tomé, E. W. Lang

**Affiliations:** 1 CIML, Biophysics, University of Regensburg, 93040 Regensburg, Germany; 2 Department of Radiation Oncology, Universitätsklinikum Erlangen, Friedrich-Alexander-Universität Erlangen-Nürnberg, 91054 Erlangen, Germany; 3 IEETA, DETI, Universidade de Aveiro, 3810-193 Aveiro, Portugal; 4 Center for Medical Physics and Engineering, University of Erlangen-Nuremberg, 91052 Erlangen, Germany; US Department of Agriculture, UNITED STATES

## Abstract

During High Dose Rate Brachytherapy (HDR-BT) the spatial position of the radiation source inside catheters implanted into a female breast is determined via electromagnetic tracking (EMT). Dwell positions and dwell times of the radiation source are established, relative to the patient’s anatomy, from an initial X-ray-CT-image. During the irradiation treatment, catheter displacements can occur due to patient movements. The current study develops an automatic analysis tool of EMT data sets recorded with a solenoid sensor to assure concordance of the source movement with the treatment plan. The tool combines machine learning techniques such as multi-dimensional scaling (MDS), ensemble empirical mode decomposition (EEMD), singular spectrum analysis (SSA) and particle filter (PF) to precisely detect and quantify any mismatch between the treatment plan and actual EMT measurements. We demonstrate that movement artifacts as well as technical signal distortions can be removed automatically and reliably, resulting in artifact-free reconstructed signals. This is a prerequisite for a highly accurate determination of any deviations of dwell positions from the treatment plan.

## Introduction

High dose rate brachytherapy (HDR-BT) recently became an alternative to whole breast irradiation of tumors in female breasts during radiation therapy [1, 2]. Thereby roughly 14 − 25 catheters are inserted into the female breast by a surgical intervention [3]. During radiation treatment, then a radioactive source, ^192^*Ir* for example, is moved inside the catheters following a treatment plan [4, 5] which fixes dwell positions and dwell times. The latter are designed to assure an optimal and intense irradiation of the tumor bed while, at the same time, keeping side effects, i. e. irradiating healthy tissue, at a minimum. This strategy follows from the observation that tumor recurrences most frequently happen at the tumor bed. HDR brachytherapy commonly utilizes a remote afterloader unit to deliver high activity ^192^*Ir* radioactive source, radiating 380 [*keV*] *γ*-photons directly into the tumor treatment volume. Application of such high radiation doses (5 − 35 [*Gy*]) affords ensuring accurate radiation dose delivery in accord with the treatment plan. Such high positioning accuracy requires keeping the catheters in the same position during all subsequent fractions, so as to precisely deliver the planned radiation dose. Given these constraints, it is mandatory to assure the highest precision in source positioning according to the treatment plan. The latter is deduced from X-ray computed tomography (CT) images which are taken after the cancerous tissue has been removed by a surgical intervention (Lumpectomy). Quality assurance thus affords that the exact spatial localization of the radiation source relative to the anatomy of the patient, and its concordance with the treatment plan, needs to be determined before the treatment starts.

Implant geometry, hence indirectly radiation source localization, can be determined with an electromagnetic tracking (EMT) device. Thereby a field generator (FG) produces a magnetic field extending across the female breast. The EMT system used in this study applies a three-pole transmitter to generate an electromagnetic field. Inside the catheters then a five degrees of freedom (5DoF) solenoid sensor is moved in accord with a pre-defined treatment plan. The sensor induces a distance—dependent induction voltage which allows to determine its spatial position (dwell position) and mean residence time (dwell time).

The precision of the EMT system itself, and the accuracy of the registration between the field generator (FG) (EMT data set) and the CT imaging (Digital Imaging and COmunications in Medicine (DICOM) data set) coordinate systems, have a strong impact on the performance of an EMT system in HDR-BT catheter reconstruction. Performance assessments of EMT systems are mostly done with ideal, undistorted laboratory settings intended to mimic a clinical BT treatment environment. Corresponding data from a clinical environment are almost non-existent. Recent reviews by [6, 1, 2] discuss quality assurance aspects, catheter reconstruction accuracy and catheter technology in HDR-BT. Especially [1] reports a typical accuracy of 0.9 ± 0.2 [*mm*] which could be obtained for dynamic tracking in HDR-BT when using an optimal, distortionless, i. e. phantom-based, configuration and a pulsed DC EMT system. With an AC EMT system, ideally an accuracy of 0.26 ± 0.16 [*mm*] was achieved [7]. The error increased to more than 2 [*mm*] when magnetic field distorting equipment (a 20 *inch* Liquid Crystal Display (LCD) monitor) was approaching 30 [*cm*] of the center of the magnetic field generator (FG). A recent study by [8] used EMT, in combination with a rigid coherent point drift (CPD) algorithm [9], to estimate relative positions of a five Degrees-of-Freedom (5DoF)—sensor which was manually inserted into the catheters. Additionally, in combination with fiducial 6DoF—sensors placed onto the surface of the breast, the influence of the breathing motion has been compensated. However, objections concerning the reliability of such corrections within a long term treatment have been raised in [2]. Nevertheless, the authors claim an achieved average deviation of 1.3 [*mm*] on the registered implant geometry on the CT—couch. The corresponding accuracy of the EMT—based dwell position determination resulted in a mean deviation of 2.4 [*mm*] relative to the treatment plan. A further recent report using EMT for catheter reconstruction in HDR-BT achieved 0.7 [*mm*] in positioning and 0.2deg in orientation [5]. This study also avoided external registration by using an iterative closest point algorithm employing a finite difference method to compare reconstruction results from both conventional CT and EMT.

Data from an EMT system is based on the coordinates of the field generator, i. e. on *C*_*FG*_. This data has to be transformed to the treatment planning coordinate system *C*_*TPS*_. Note that in general this transformation is highly anisotropic. A rigid-body, point-based registration is often conducted based on three or more fiducial points with positions known in both coordinate systems through optical measurements [10], [11]. Thus the system yields the coordinates of the fiducial points which are either exported from the treatment planning system or are received as electromagnetic FG coordinates. This combination of EMT and imaging system adds up uncertainties from both modalities, thus yields errors in catheter reconstruction much larger than corresponding intrinsic EMT tracking errors [1]. By registering EMT and CT dwell positions, the residual mean error per catheter was found to be 0.6 ± 0.2 [*mm*], with a maximum catheter error of 0.9 ± 0.4 [*mm*] and a maximal dwell position error of 1.3 ± 0.7 [*mm*]. Also catheter swaps and catheter tip shifts could be detected with high sensitivity and specificity [12]. Field distortions based on nearby metal parts can only be corrected by calibration procedures which imply a fixed environment and preparatory measurements [13]. Again, error measures are based on a phantom study, while real clinical applications are very scarce [14]. Commonly the radiation treatment encompasses several sessions which extend over a whole week, roughly. Between the sessions, the patients move around which caries the risk for any spatial displacement of the catheters away from their positions deduced from the CT—image and prescribed in the treatment plan. To quantify such displacements only from EMT measurements, we recently [15] proposed a method based on multi-dimensional scaling (MDS) techniques [16], [17], [18].

An additional complication arises as patients are breathing during the EMT measurement, and if they are also speaking, the resulting breathing signal can become highly complex and erratic. To aid removing such motion artifacts from the EMT recordings, in this proof-of-principle study additional fiducial sensors are fixed on the chest of the patient. Their spatial positions are also determined via an EMT measurement. Last but not least, occasional device malfunctions add further artifact signals to the EMT recordings. In a recent study [19] we proposed a new way to get rid of such movement artifacts suggesting to employ an ensemble empirical mode decomposition (EEMD) of both the fiducial sensor and solenoid sensor signals combined with particle filtering techniques. Particle filters [20], [21] represent sequential Monte Carlo techniques and are well known from applications to system identification problems. They are employed here to precisely track the trajectory of the solenoid sensor inside the catheters without any noise contributions. The measured spatial dwell positions inside the catheters provide source trajectories which, ideally, should be in perfect agreement with the treatment plan. However, patient movements between the treatment sessions and breathing motions during the EMT measurements cause signal distortions which result in deviations from the dwell positions defined in the treatment plan. As we will show, such deviations can be precisely quantified after the measured EMT signals have been tracked with a particle filter and superimposed breathing mode artifacts have been removed with the help of an EEMD analysis.

System identification techniques have hardly been applied to HDR-BT data since. A recent study [22] considered sensor tracking with an extended Kalman filter. It was mainly devoted to achieve a dynamic field distortion compensation within an EMT measurement. The authors used additional redundant sensors and a realistic state evolution model. They applied the SLAM algorithm [23], [24] and combined it with an extended Kalman filter to be used in pre-calibration procedures for system identification.

The currently proposed protocol combines particle filter tracking methods with multi-dimensional scaling thus enabling the exclusive use of intrinsic EMT measurements of spatial positions of the solenoid sensors for catheter reconstruction. In addition, it relies on empirical mode decomposition techniques to decompose the recorded non-stationary time series and remove breathing artifacts in real clinical applications with patients. The newly proposed method robustly and almost perfectly reconstructs the shape of various catheters involved in any HDR-BT treatment, quantifies any deviations from the treatment plan and conveniently visualizes the dwell position tracks of both, the CT data set and the various EMT data sets.

The manuscript is organized as follows: Section 1 provides an *Introduction* and a discussion of related work. In Section 2, entitled *Materials and Methods* we discuss details of the *Data Acquisition* process and proposes a new *Data Analysis Methodology* based on a singular spectrum analysis (SSA) for denoising and high amplitude measurement artifact removal followed by an EEMD to isolate and identify the breathing mode contribution and remove it from the signal. We also provide details of the application of the various data analysis techniques proposed in this proof-of-principle study. Section 3 presents the *Results* and offers a *Discussion* of those findings. Finally, Section 4 draws some *Conclusions*. Most of the more technical material is, however, put into Appendices for the convenience of the reader.

## Material and methods

### Patient cohort

This investigation is intended to serve as proof-of-principle of the proposed automatized data processing chain. Hence it relies on data that was collected in a recent study from four female patients with an age of 49 − 68 years. All women have been thoroughly informed and have given their written consent to the treatment which, furthermore, has been approved by the institutional review board of the Friedrich-Alexander-University Erlangen-Nürnberg (*Nr*. 355 − 14*B*, 2014). The study comprised 4 patients between 49 − 68 years of age. The women underwent a high dose rate brachytherapy treatment of a breast cancer that was excised in a surgical intervention (Lumpectomy) before the radiation treatment. For the HDR-Brachytherapy radiation treatment, catheters were implanted in the breast of the patients following a pre-defined geometry designed by a experienced radiologist. The number of catheters varied from case to case with a mean number of implanted catheters of 18.25. Based on an initial X-ray computed tomography (CT), a treatment plan was created with an Oncentra program (Oncentra^®^ Brachy v4.3, Elekta, Veenendaal, The Netherlands) which stores all data in the Digital Imaging and Communications in Medicine (DICOM) format. The contours for the treatment plan were defined by a medical expert according to a prescription given in [25]. For each catheter, a different number of dwell positions was defined in the treatment plan. The mean number of dwell-positions per patient was 633.5. Altogether, for each patient, only the first session of EMT data collection was recorded on the CT bench. This imaging data was used for treatment planning and formed the reference data set to which all EMT-based data sets had to be compared. All subsequent EMT measurements then have been performed in an HDR Brachytherapy treatment room on a specifically prepared patient bench.

### Image acquisition and treatment planning

Computed tomography X-ray images (CT image) have been acquired either with breathing command, where an X-ray image is recorded while patients stopped breathing. For the treatment plan, the catheters were reconstructed from CT image slices by a medical physicist. Hence, the reconstruction of the catheters depends on experience and attention of the medical physicist. This data set is designated as CT data set.

The catheters were implanted on the first day of the treatment period. Before the CT is acquired, the catheters are cut to a length of 287.5 [*mm*]. During treatment, the sensor is inserted into the catheter to its very end. From the very end of the catheter, a default margin of *l*_0_ = 5 [*mm*] is left. There the first dwell-position is set, and all subsequent dwell positions are chosen in steps of 2.5 [*mm*]. Not all these dwell positions are set active in the treatment plan, however. Each dwell position is numbered as well as each catheter. From the EMT measurements, the dwell-positions are calculated with the raw EMT data according to a method proposed in [26].

### EMT recordings

The dwell positions of a 5—DoF sensor, introduced into each catheter consecutively, were determined by a *Flexitron^®^* (Electa AB, Stockholm, Sweden) afterloader with electromagnetic tracking capabilities and relied on a measurement software based on the rigid coherent point drift (CPD) algorithm as detailed in [26]. The Flexitron system comprises a treatment delivery unit (TDU), treatment control panel (TCP) and a treatment communication console (TCC). The system control (SCU), field generator (FG) and the sensor interface units (SIU) jointly constitute a prototype of an NDI Aurora^®^ (Northern Digital Inc., Canada) EM tracking system. The measurement data is collected by a computer. The catheters are connected with the afterloader via transfer tubes of total length 1000 [*mm*]. The length inside each catheter for the source applicator is 400 [*mm*]. The speed of the sensor can be varied between 2.5 [*cm*/*s*] − 50 [*cm*/*s*] [27]. The smallest step size between two dwell positions amounts to Δ*s* = 1 [*mm*].

For an EMT measurement, a field generator (FG) is connected with a tracking system and placed above the chest of the patient. During the automatic movement of the EM sensor along the inside of the catheter within the field of view of the field generator, the spatial coordinates of the sensor can be derived via induction voltage signals generated in the solenoid sensors. The distance between FG and the sensor critically influences the signal sensitivity. Additionally, three 6 DoF fiducial sensors are placed on the thorax of the patient to measure the breathing motion. All data are made available at https://osf.io/kd6ta, DOI: 10.17605/OSF.IO/KD6TA for the convenience of the reader.

#### Phantom data

The catheters implanted into the phantom were connected to the afterloader via transfer cables. The treatment plan defined for the phantom was up-loaded to the software of the Flexitron^®^ afterloader prototype (Electa AB, Stockholm, Sweden). The sensor was moved to each dwell position inside the catheters according to the treatment plan and remained at the dwell positions for the defined dwell time.

**Fig 1:**
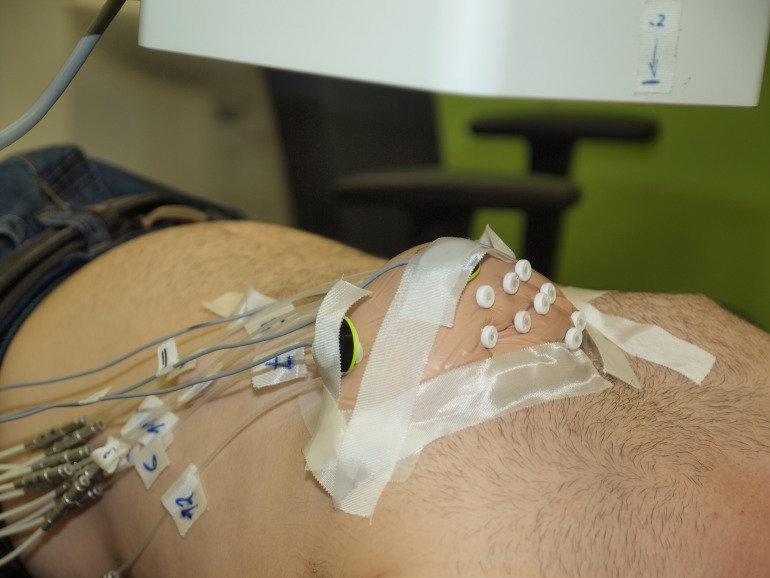
Breast prosthesis with 12 implanted catheters fixed to the chest of a male test person. To monitor the breathing motion, three fiducial sensors are fixed to the breast.

To investigate the possibility of a breathing compensation, preparative measurements with a phantom have been performed. 12 catheter tubes were implanted into a breast prosthesis by a surgeon (see Fig 1). The template and needles from the radiation surgery were used for implantation. The tubes were cut to a length of 287.5 [*mm*]. A computer tomography (CT) X-ray image of the breast phantom was acquired. Also a treatment plan was created based on the catheters’ shapes in the CT image. In a subsequent experiment, the breast phantom was carefully fixed to the chest of a male test person. With this design, some very small catheter displacements could arise from occasional spatial displacements of the prosthesis. However, they were smaller than the precision of EMT measurement. The phantom was measured three times according to the following protocol: the person was either

- breathing calmly in the chest, or
- breathing calmly in the belly, or
- breathing while speaking.

The measured sensor movement then consisted of a superposition of the chest motion and the sensor motion. Thereby, the chest motion was in a direction roughly perpendicular to the sensor motion inside the catheters.

#### Patient data

After the preparatory phantom measurements, data measured from four patients was analyzed. Depending on the location of the tumor, in each of the patients, the catheters were implanted in different anatomical planes. A patient specific treatment plan was defined for each of the four patients by an experienced medical physicist. For each of the patients, EMT data was recorded in an HDR Brachytherapy treatment room on a specifically prepared patient table. The movement of the solenoid sensor according to the treatment plan was tracked with the NDI Aurora^®^ (Northern Digital Inc., Canada) EM tracking system in combination with the Flexitron^®^ (Electa AB, Stockholm, Sweden) afterloader system. The measurements were performed in analogy with the phantom case. The first measurement took place one day after the surgical implantation of the catheters. Though the field generator axes remained always the same, the implant plane influences the spatial direction of the highest breathing amplitude. Two of the patients were speaking during the measurement while the other two were breathing calmly. The success of the reconstruction of the catheters’ shapes without the breathing mode contribution strongly depends on the amplitude of the breast movement during breathing and/or speaking. The women analyzed in this study differed in the number of implanted, and finally used, catheters, their breast volumes and their ages. In Table 1, all relevant information about the patients and their treatment plans is collected [26].

**Table 1:** Summary of all patient data and treatment plan variables for all measurements conducted in this study.

Patient	age	impl. cath.	used cath.	dwell pos.	implantation plane
phantom	–	12	8	367	transversal
01	49	14	12	505	sagittal
02	51	17	11	547	sagittal
03	68	19	15	540	transversal
04	62	23	20	942	sagittal and transveral

### Data analysis methodology

Following we describe a data analysis methodology to automatically analyze the electromagnetic tracking (EMT) measurements obtained during a HDR-BT treatment. The induction voltage signals are obtained from a solenoid sensor moving inside various catheters implanted into the breast of female patients, and three fiducial sensors fixed to the chest of the patient, respectively. The movement of the 5 DoF solenoid sensor in the inhomogeneous magnetic field of a field generator is characterized by the sensor’s dwell positions. An independent measure of the breathing mode is obtained with EMT measurements of signals from the fiducial sensors.

Such measurements contain a number of artifacts, mainly those from the breathing motion of the patients during the measurement. The proposed *data processing chain* encompasses the following steps:

- *Sensor tracking*: A *particle filter* (PF) technique is employed to track the dwell positions of a solenoid sensor inside various catheters implanted in a female breast for a radiotherapy treatment.
- *Artifact removal*: Next, *singular spectrum analysis* (SSA) and *ensemble empirical mode decomposition* (EEMD) are applied to remove artifacts from the recorded signals.
- *Dwell position deviations*: Finally, a *multi-dimensional scaling* technique is employed to quantitatively compare sensor dwell position measurements recorded with respect to different coordinate systems.

#### Goal of the study

Up till now, all these signal pre-processing techniques are done interactively and need the intervention of the user. This is a tedious procedure. Hence the goal of the present study is to automatize this data analysis and to establish a data analysis protocol without recourse to any external registration of the breathing motion by other methods than EMT. Static and dynamic experiments on a Phantom serve to establish the methodology and to prove the robustness and reliability of our data analysis methodology. Additionally a human medical expert is asked to check all results and approve their correctness. Thus we want to provide an objective and reliable data analysis tool to automatically, efficiently, precisely and reliably evaluate EMT measurements recorded during a HDR-BT

The application of PF [19] and MDS [15] has been described recently, hence only a short account will be provided here. The current study rather focuses on automatic artifact removal techniques based on SSA and EEMD. We will show that a combination of both methods is able to remove noise and breathing mode contributions superimposed onto the EMT sensor signals.

Generally, the EMT sensor signal is ballasted with measurement noise. To estimate the true sensor state during the sensor’s motion inside a catheter, we recently proposed to employ a *particle filter* to estimate the underlying but unobservable sensor state from the noise contaminated observation of the dwell positions provided by the EMT system.

However, particle filtering cannot remove large amplitude artifacts occasionally observed on bothe the solenoid sensor and the fiducial sensor signals. Here we suggest to apply SSA to remove such artifacts.

Because patients breath during the EMT measurements, the recorded EMT signal is corrupted by the superimposed breathing mode contribution and eventually occurring additional motions of the patients lying on the treatment table. In case, patients are speaking during the measurements, these breathing motions can become highly irregular. To automatically remove such breathing artifacts from the EMT solenoid sensor signals, we propose to decompose both the fiducial and the solenoid sensor signals with an EEMD, identify those intrinsic modes which are dominated by the breathing modes, and reconstruct the sensor signal neglecting such contaminating modes.

The automatic identification of intrinsic modes underlying the measured solenoid sensor signal via a comparison with the intrinsic modes of the fiducial sensor signal is achieved with a proper similarity measure. Several such measures have been tested with rather similar results.

Finally, EMT dwell position measurements from subsequent treatment sessions of the sensor inside the catheters refer to different coordinate systems. Hence, results are not comparable straighforwardly. Recently we proposed a multi-dimensional scaling scheme to rectify this mismatch, thus rendering different dwell position measurements quantitatively comparable. Thus an MDS is finally applied to the artifact-free dwell position tracks resulting from particle filtering.

We demonstrated the principle usefulness of this data analysis strategy which does not need any additional external registration. We also corroborated a robust and reliable identification of all sensor dwell positions, and their deviations from the treatment plan, during an EMT measurement to identify and characterize the shape, i. e. the spatial location and orientation, of all implanted catheters used during the radiation treatment.

**Fig 2:**
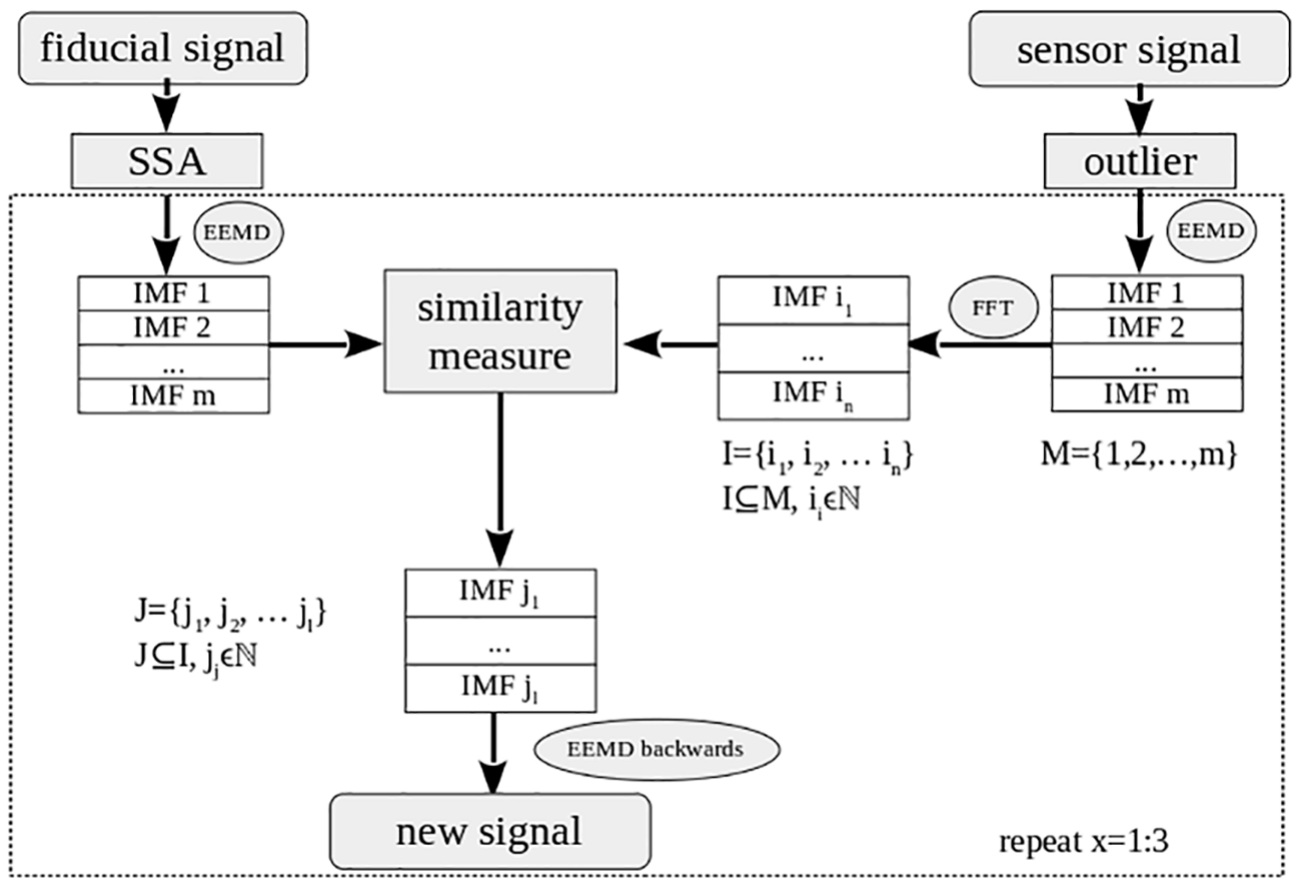
Flowchart of the algorithm employed in the artifact removal step of the data processing chain.

In Fig 2, the structure of the data processing chain is illustrated in a flowchart. The goal of this data analysis methodology is to obtain the shape of the catheters and the spatial coordinates of the dwell positions, where the sensor stays a defined dwell time. In addition, three fiducial sensors are placed on the patient’s chest to measure the breathing signal. The sensor, inserted into the implanted catheters, instead measures a superposition of the breathing motion and its movement along the catheter.

#### Measurement protocol

Sensor tracking with sequential Monte Carlo techniques: In system identification problems, state space models with latent state variables are used to estimate underlying dynamic systems states from noise-corrupted measurements. Bayesian state estimation is achieved by combining such observations with prior knowledge of the physical system to sequentially estimate the underlying unobservable system states in a statistically optimal way. Referring to EMT measurements in HDR-BT, the observations correspond to sensor dwell positions $z_{m}^{\left(p,r\right)},p=1,\ldots ,P,r=1,\ldots ,R$ measured at discrete times *t*_*m*_ inside catheter *p* during treatment session *r*. The latent states correspond to the underlying exact spatial positions $x_{m}^{\left(p,r\right)}$, given some physical model of the sensor motion. To simplify the treatment, several Markovian approximations are generally applied neglecting any memory effects as well as cross-dependencies of the variables [21]. The underlying theory of particle filters is shortly summarized in Appendix 1.

Artifact removal with singular spectrum analysis: To achieve this goal, first of all, the noise contribution to the fiducial signal and the measurement artifacts in the sensor signals due to technical malfunction have to be removed. Here we propose to employ *Singular Spectrum Analysis* (SSA) [28], [29] to achieve denoising and artifact removal [30], [31] either from the fiducial or the sensor signals. For the convenience of the reader, a short account of SSA is presented in Appendix 2.

**Fig 3:**
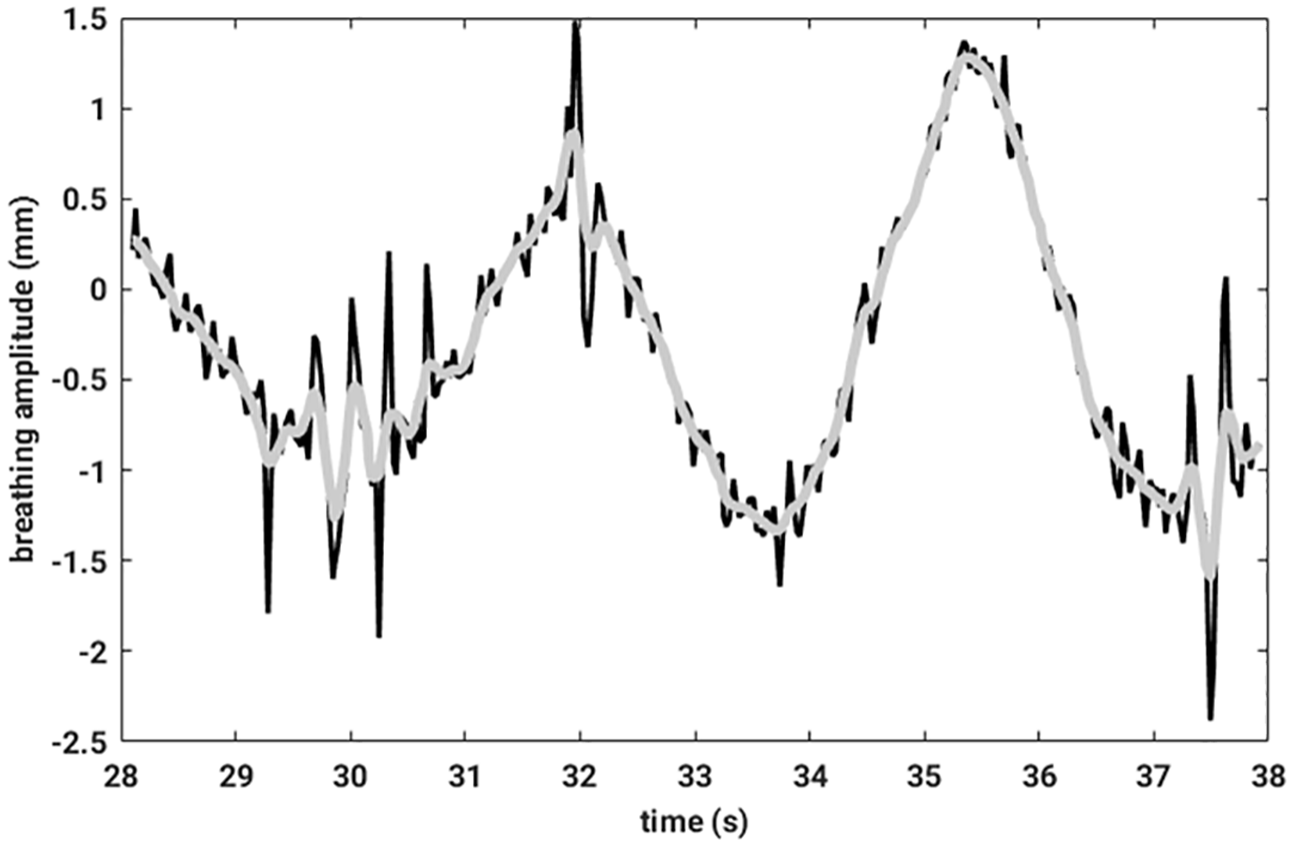
The measured raw breathing signal is colored in black, and in grey the first component of the SSA is depickted.

**Fig 4:**
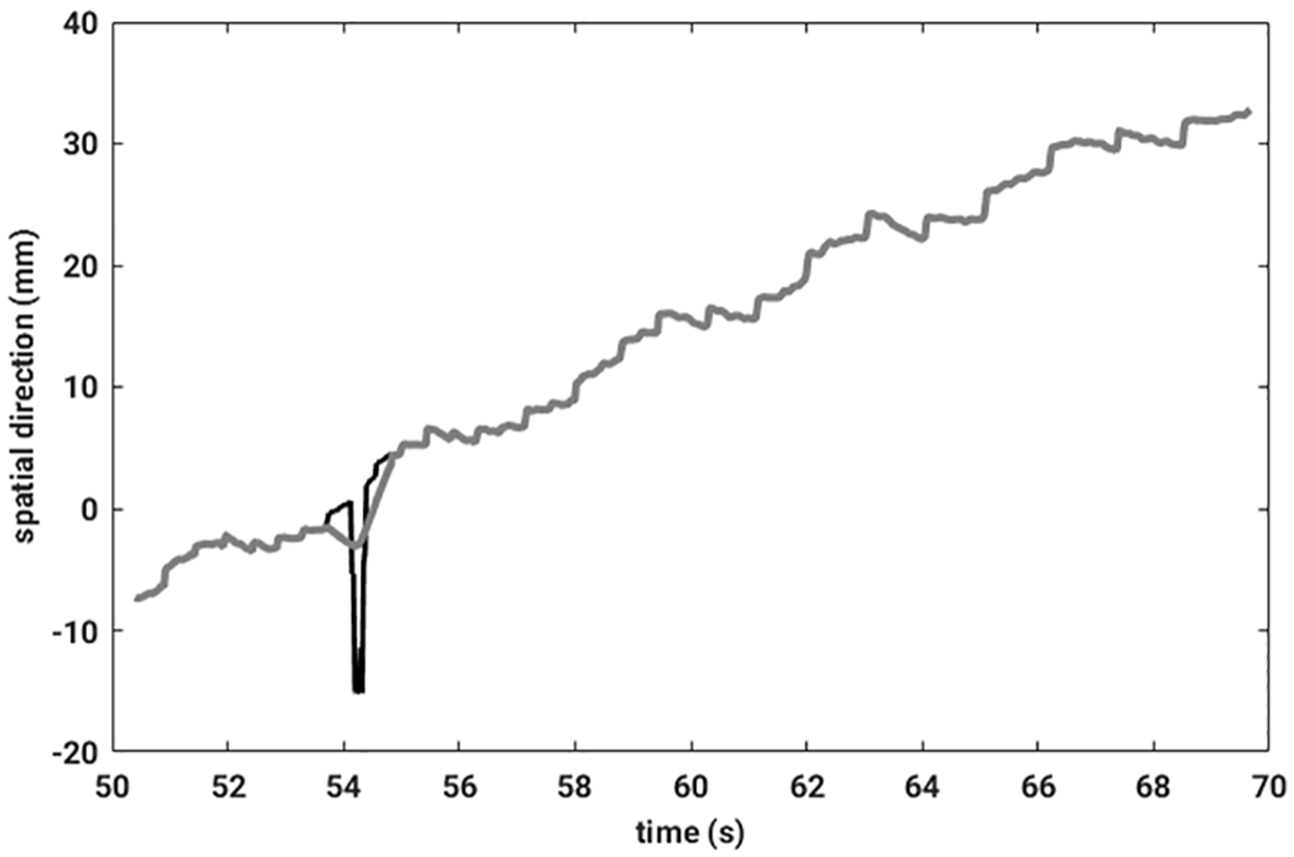
The measured raw sensor signal is drawn in black, and a grey line illustrates the signal resulting after outlier removal.

- Fiducial signal
  First of all, an average breathing signal was calculated by averaging over the three fiducial signals. This signal was not tracked by a particle filter. Consequently, it was very noisy. To get rid of the noise on this signal, an SSA was applied to the latter. For further computations, only the first SSA component was considered [32]. In Fig 3, the original mean breathing signal and the first component of an SSA decomposition is compared.
- Outlier detection
  Despite a particle filter tracking of the sensor signal, the data still contained outliers due to technical malfunctioning of the EMT measurement apparatus. If, for technical reasons, a measured value is missing, the software puts zeros as entries of the data records. Employing particle filter tracking, such anomalous measurements will then lie closer to the regularly measured points but still will not lie on a smooth line. Therefore, data needs to be preprocessed to remove such outliers. To identify any measurement point as outlier, the following condition had to be fulfilled: Let *x* be the signal measured in one spatial direction, then
  $$\begin{array}{c} \left|x-median(x)|>2\cdot std(x\right) \end{array}$$
  Afterwards, an SSA can be applied to the sensor signal. Instead of taking only the first component for further calculation, as in the case of fiducial signals, only the outliers and their neighboring data entries were replaced by the corresponding entries from the first SSA component. In this way information about the stop positions could be preserved. An illustrative example of an outlier removal is shown in Fig 4.

Artifact removal with an ensemble empirical mode decomposition: After the signal is corrected from noise and outliers, there is still the breathing signal superimposed onto the EMT sensor signal reflecting its movement inside the catheter. The primary goal of this study is to automatically remove this movement artifact with the help of an *empirical mode decomposition* (EMD) [33], [34] and reconstruct the precise shape of the catheter. An EMD [35] can be applied to any non-stationary and non-linear time series to extract intrinsic modes which locally represent pure oscillations with varying amplitude and local frequency. Its noise-assisted ensemble variant EEMD [34] avoids mode mixing and boundary artifacts.

**Fig 5:**
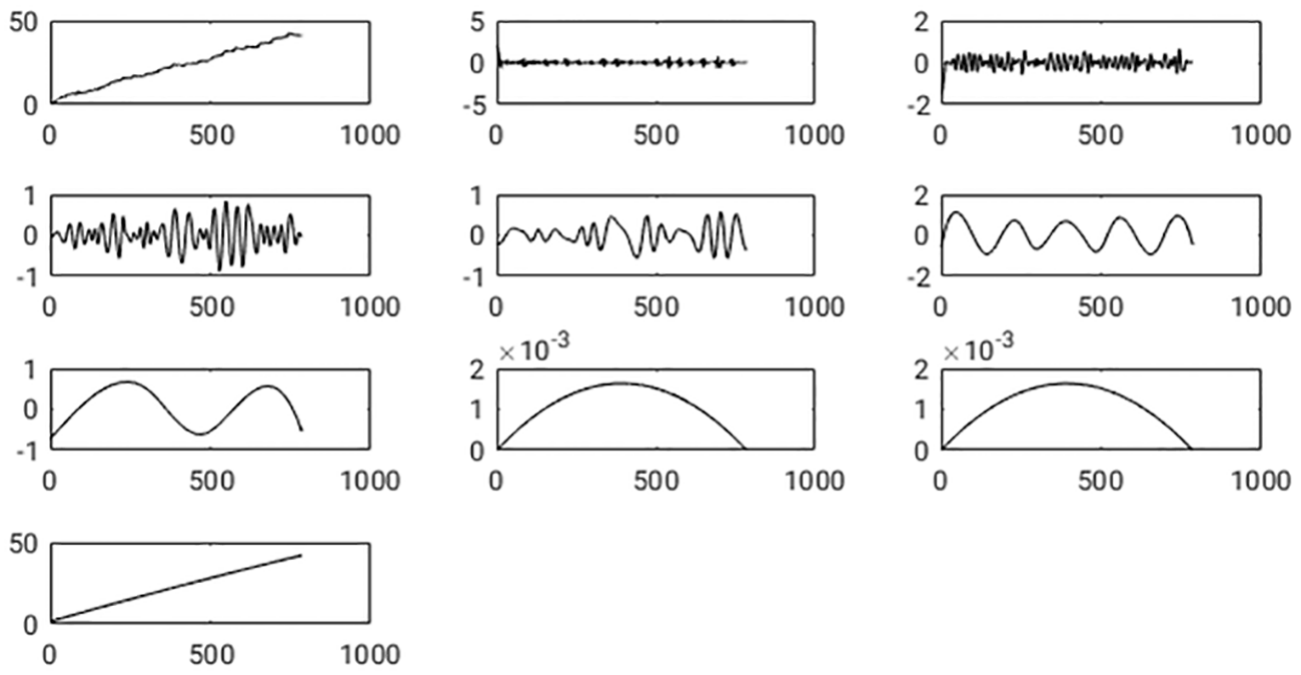
Shown is the spatial sensor position along one coordinate axis versus discrete time *n* ⋅ *T*_*s*_ with *T*_*s*_ the sampling interval and *n* = 1, …, *T*. In the left corner of the top row, the original signal is exposed. The other boxes show the IMFs resulting from an EEMD. On the x-axis of each graph, the number of samples, and on the y-axis, the signal amplitude is plotted. The first IMF comes aside of the original signal, and subsequent IMFs follow in each row from left two right.

To get rid of the breathing artifact, in the current study, an EEMD is applied to the solenoid sensor signal to extract its underlying intrinsic modes (IMFs). The EEMD is also applied to the principal component of the average signal deduced from the fiducial sensors. As EEMD is known to obey the full reconstruction property, the idea obviously is to reconstruct the signal from its components while neglecting the component representing the breathing mode. The decomposition of the EMT sensor signal was done within an ensemble *E* where each time a different noise contribution is added during the sifting process. In this study, the size of the ensembles was set to *E* = 10. The results did not change if a larger size was chosen. Note that the IMFs are sorted naturally by their dominant frequency. Consequently, the highest frequency is in the first IMF, and the last IMF only represents a non-oscillating trend in the time dependence of the sensor signal. In Fig 5, the original sensor signal and all IMFs resulting from an EEMD are shown.

**Fig 6:**
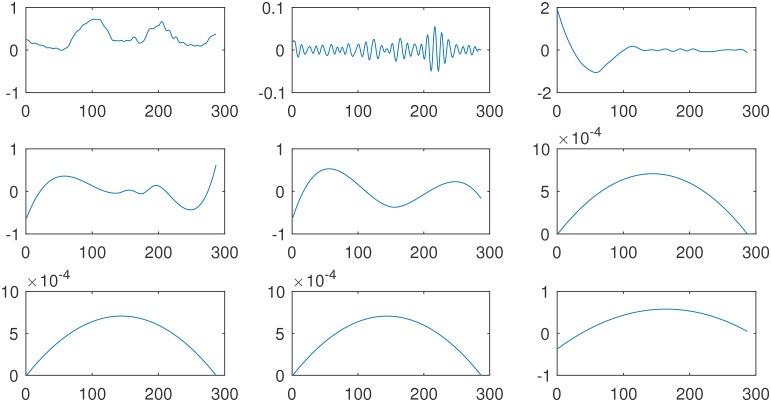
Shown is the spatial fiducial sensor position along one coordinate axis versus discrete time. In the left corner of the top row, the original signal is exposed. The other boxes show the IMFs resulting from an EEMD. On the x-axis of each graph, the number of samples, and on the y-axis, the signal amplitude is plotted. The first IMF comes aside of the original signal, and subsequent IMFs follow in each row from left two right.

**Fig 7:**
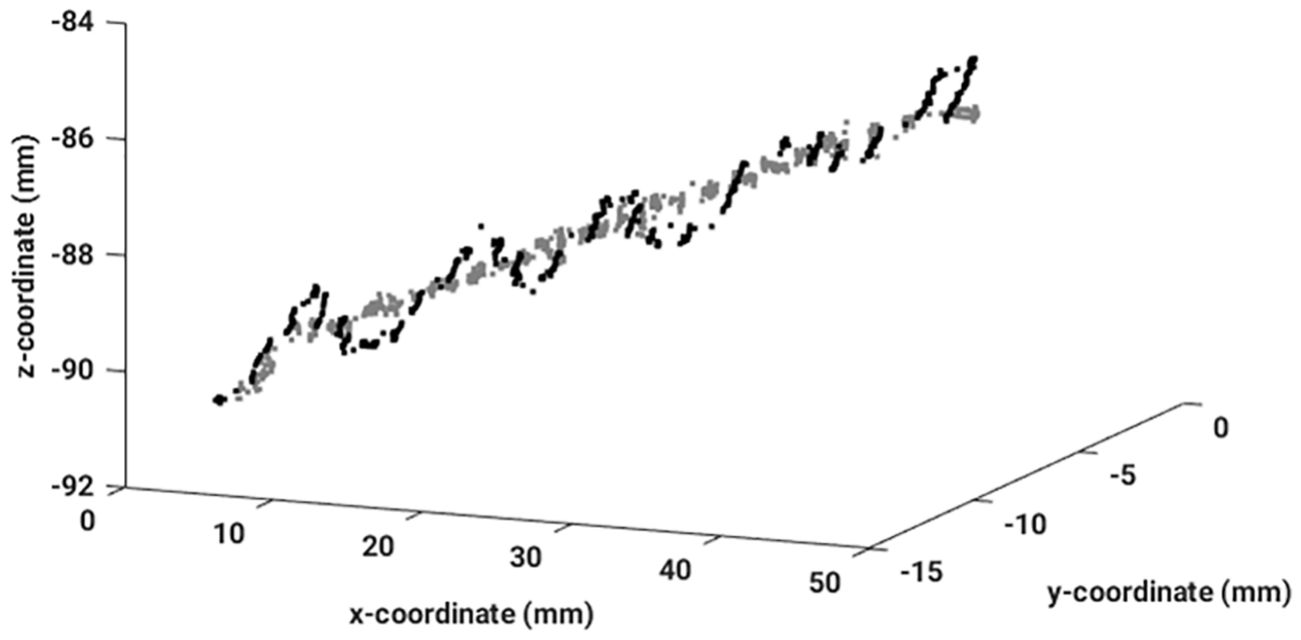
The originally measured EMT signal from one catheter (black), and its reconstructed counterpart (grey) are shown to illustrate the efficiency of the removal of the breathing mode contamination.

As a next step, the IMFs which contain the breathing signal had to be identified. To achieve this, the EEMD also has been applied to the principal component of the breathing signal measured with the fiducial sensors. The resulting IMFs are illustrated in Fig 6. If the IMFs from the two signals are compared with each other visually, one recognizes that IMF *c*^(3)^ from the breathing signal looks very similar in frequency content and spatial shape to IMF *c*^(5)^ from the sensor signal, as well as IMF *c*^(4)^ (breathing) and IMF *c*^(6)^ (sensor). To achieve an automatic assignment of corresponding intrinsic modes from both signals, in this study the identification of the IMF which is similar to the breathing signal is performed employing different similarity measures. Afterwards, the sensor signal was reconstructed while neglecting the contaminating IMFs. In Fig 7 one catheter is shown, where the breathing motion was removed along each spatial direction separately.

A crucial point during reconstruction, however, is not to loose components containing information about the sensor dwell positions. Thus, a *Fast Fourier Transformation* (FFT) is applied to each IMF from the sensor signal to identify the IMFs containing information about the dwell positions. These IMFs cannot be neglected during reconstruction. Having achieved this, the sensor signal is reconstructed ignoring those IMFs identified to contain contaminations from artifact signals. This procedure can be repeated several times. Clearly, the methods work very efficiently, and the breathing signal could be suppressed completely. Appendix 3 summarizes the theory of EEMD for the convenience of the reader.

Similarity measures: In the course of this study, sensor signals, measured with an EMT device to follow their path inside any of the catheters implanted in the female breast, are decomposed employing an EEMD (see [19] for details). Much the same happens to the signals collected from fiducial sensors which mainly monitor any breathing motion of the patients during the EMT measurements. The goal of this procedure is to identify those intrinsic modes of the sensor signals which most closely resemble the breathing mode artifact. During signal reconstruction, these artifacts then will be removed from the superposition of the intrinsic modes to reconstruct an artifact-free sensor signal. To achieve this goal, proper similarity measures [36], [37] between time series need to be explored. Hence in Appendix 4 we will discuss several alternative measures of similarity that have been applied to the decomposed sensor signals.

Multi-dimensional scaling: In this study, MDS finally serves to jointly map positional information contained in the CT—image as well as in the EMT measurements, after these data is pre-processed in the way sketched above. First, an X-ray CT—image is taken after surgical implantation of the catheters into the female breast, from which a treatment plan is established and dwell positions and dwell times are defined. Subsequently, sensor dwell position measurements are performed during the EMT sessions, which extend over a whole week mostly. Because of changing relative positions of the female patient in the magnetic field of the field generator of the EMT system, each sensor tracking measurement refers to a different coordinate system. MDS is a shift-invariant technique which only relies on distance information. It estimates a common principal coordinate system which best explains all observed distances. Given such a reference coordinate system, any observed deviations from the treatment plan of the various sensor dwell positions in the different catheters can be identified, quantified and conveniently visualized. The basic theory behind MDS is summarized in Appendix 5 (see [15] for further details).

Accuracy: After the breathing compensation, the spatial distribution of the measured dwell points reflects the shape of the catheters. If the sensor stopped at a dwell position for a prescribed dwell time, several sampled sensor positions happen to occur at the same spatial location. For further computations it is sufficient keeping only one value per stop position. For each measured point, the related command from the afterloader, if the sensor either stopped or moved in or out, is known by the recording system as well as the number of the dwell position. Consequently, the stop positions can be calculated by averaging over the corresponding point clouds belonging to each stop-command. Assume that *N*_*s*_ times a dwell position was measured by the EMT system at a stop position. For each dwell position, its three-dimensional coordinates, referred to the EMT-coordinate system, were collected as components of a column vector **x**_*n*_ = (*x*_1*n*_, …, *x*_*Nn*_)^*T*^, where *N* = 3, *n* = 1, …, *N*_*s*_. The dwell positions from the treatment plan, according to the CT-coordinate system, can also be summarized in corresponding column vectors. To compare the two data sets, referred to different coordinate systems, a *multi-dimensional scaling* (MDS) approach was used as is explained in detail in [15]. The MDS maps the two data sets onto a common principal component system, where one can calculate the relative deviations from the same dwell point in the two data sets. The values of these deviations were used to characterize the precision of the breathing mode compensation. Especially, the values calculated from the phantom data were important for the evaluation, because there no change of catheter shape due to tissue swelling or patient movement could occur.

## Results and discussion

### Phantom data

The preparatory measurements collected EMT sensor data recorded in a phantom fixed to a male chest. The sensors measured a superposition of the movement along the catheter and the breathing motion. EMT measurements were performed according to the following protocol:

- In the first measurement, the male proband tried to achieve a high breathing amplitude by chest breathing.
- In the second experiment, he was breathing calmly via its belly.
- In the last experiment, he was breathing while constantly speaking.

The sensor was moved according to the treatment plan, which was defined based on the X-ray CT images acquired while the phantom was fixed on the CT-table. Between the CT and the EMT-measurements, the phantom was removed from the CT-table and placed on the chest of the male proband. Therefore, some displacements could have been arisen, because of the soft material of the prosthesis. However, we carefully avoided exerting any strain onto the phantom, hence we never could observe any displacement during the EMT measurements.

The aim of this work was to remove patient movement-related signal components while precisely reconstructing the shape of the catheters from EMT measurements alone. The sensor signal as well as the breathing signal, which was measured separately on the patient’s chest via fiducials, could be decomposed by applying an EEMD. Afterwards the signal has been reconstructed by adding up all IMFs which did not contain information about the breathing signal. The human eye is very well suited to grasp similarities between two signals. Therefore, as an additional cross-check, the algorithm was applied again to all datasets, but, instead of taking a similarity measure, an experienced data analyst decided which IMF should be deleted. The goal of this visual inspection was to not delete any ‘stop’-information and to get rid of breathing artifacts. This kind of analysis created kind of a ground truth against which the performance of the fully automatic algorithm has to be tested.

**Fig 8:**
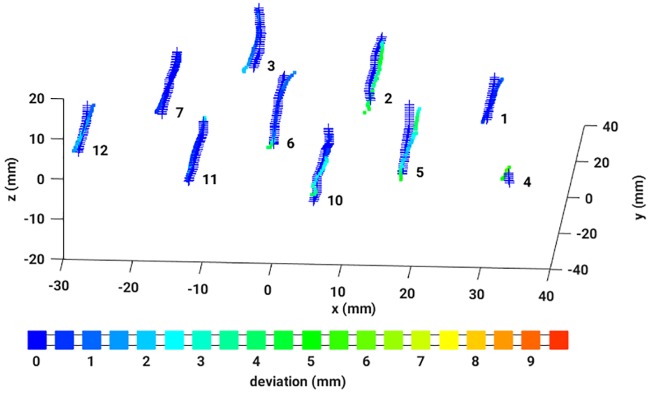
Three dimensional dwell positions from the treatment plan (crosses) and the reconstructed EMT measured dwell positions, colored according to the absolute deviation as given in the colorbar.

To have an accuracy measure, the resulting dwell positions were compared with the dwell positions defined in the treatment plan. This could be achieved by an algorithm based on MDS (see Appendix 5 and references given therein). Thus, for each pair of dwell positions, a deviation value could be assigned, and, subsequently, an average over all deviations has been calculated. In Fig 8, a reconstructed dataset is illustrated including the corresponding colorbar.

**Fig 9:**
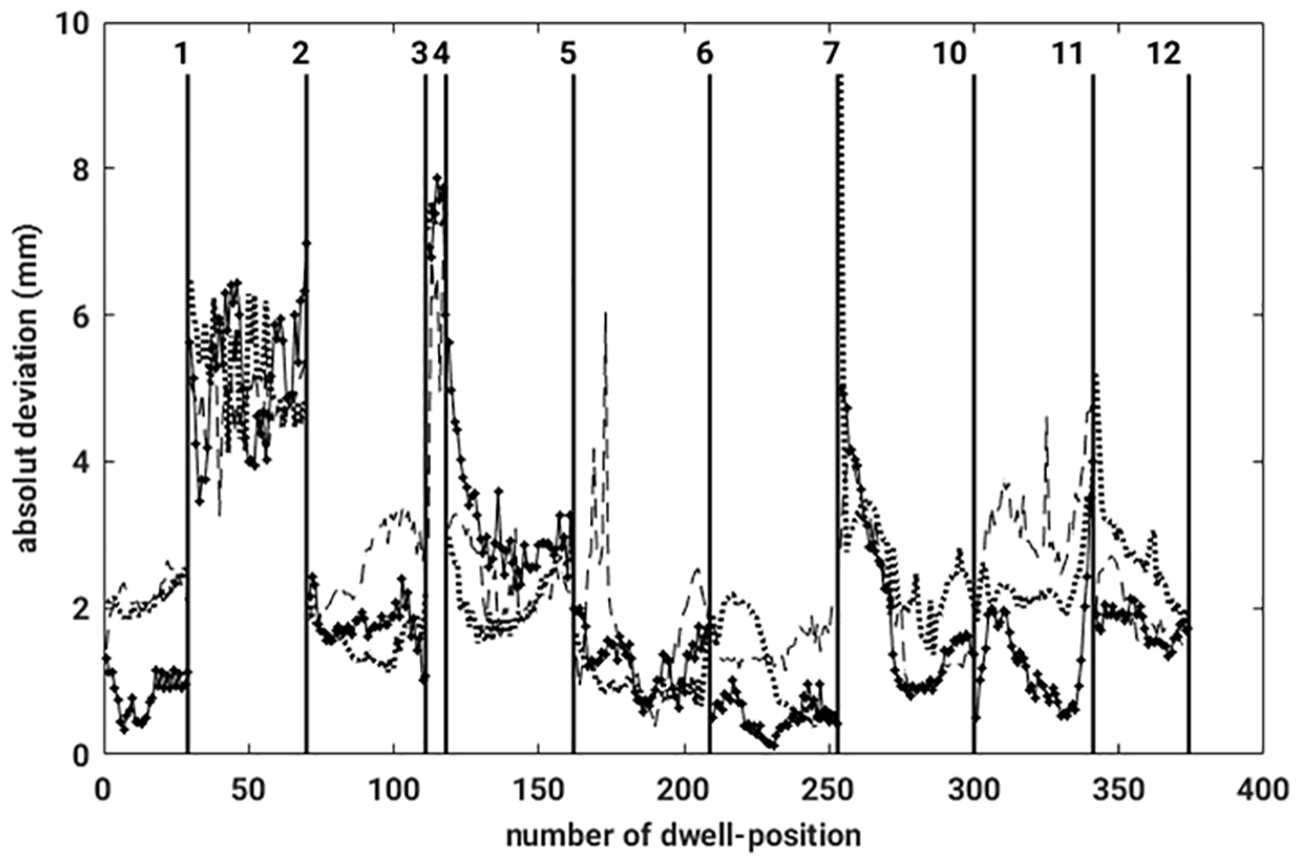
Absolute deviation for each pair of dwell positions (CT-EMT), for four different reconstructed measurements. The black vertical lines mark the separation between the different catheters.

The breathing mode was removed automatically from all three data sets obtained with the phantom. Next, a graph showing the relation between the absolute deviations and the related dwell positions has been constructed and is presented in Fig 9, displaying the results obtained for three different measurements. The vertical lines mark separate catheters, hence show which dwell positions belong to each catheter. The graph corroborates that only small differences between the three reconstructed data have been observed. The dissimilarities are in the range of the measurement uncertainty from the measurement system. The displacements between the CT and the EMT-measurements, on average, amounted to no more than 2.3 *mm*. The fact that the mean displacement values from the phantom differed from zero is because,

- on the one hand, measurement uncertainties and a high variance in the catheter reconstruction in the treatment plan exist, and,
- on the other hand, the flexible phantom was removed from the CT-table and mounted onto the male chest and fixed there, to be able to include a realistic breathing motion artifact in the EMT measurements.

Both facts contributed to a systematic but very small finite mean displacement in case of the phantom data.

The reconstructed results from all three different measurements are very similar corroborating that the algorithm works well, and also that deviations between both data sets can be quantify consistently.

### Patient data

Apart from the preparatory measurements with the breast phantom, EMT sensor data was recorded inside the catheters which were implanted into the breast of female patients.

To remove the breathing motion artifacts contaminating the patient data, much as before the phantom data, the previously described algorithm was applied to the data, and different similarity measures were applied to identify breathing mode contaminations in the IMFs deduced from the EMT sensor signal. First, the well-known Pearson correlation coefficient (PCC) was used to point-wise measure linear similarities. Next, the density—based Kullback-Leibler divergence (KLD) was employed to account for higher order correlations as well. As a third similarity measure representing a real metric, the Jensen-Shannon divergence (JSD) as a smoothed and symmetrized version of the KLD was used. Finally, the data was reconstructed also while data similarity was judged by a human expert.

**Fig 10:**
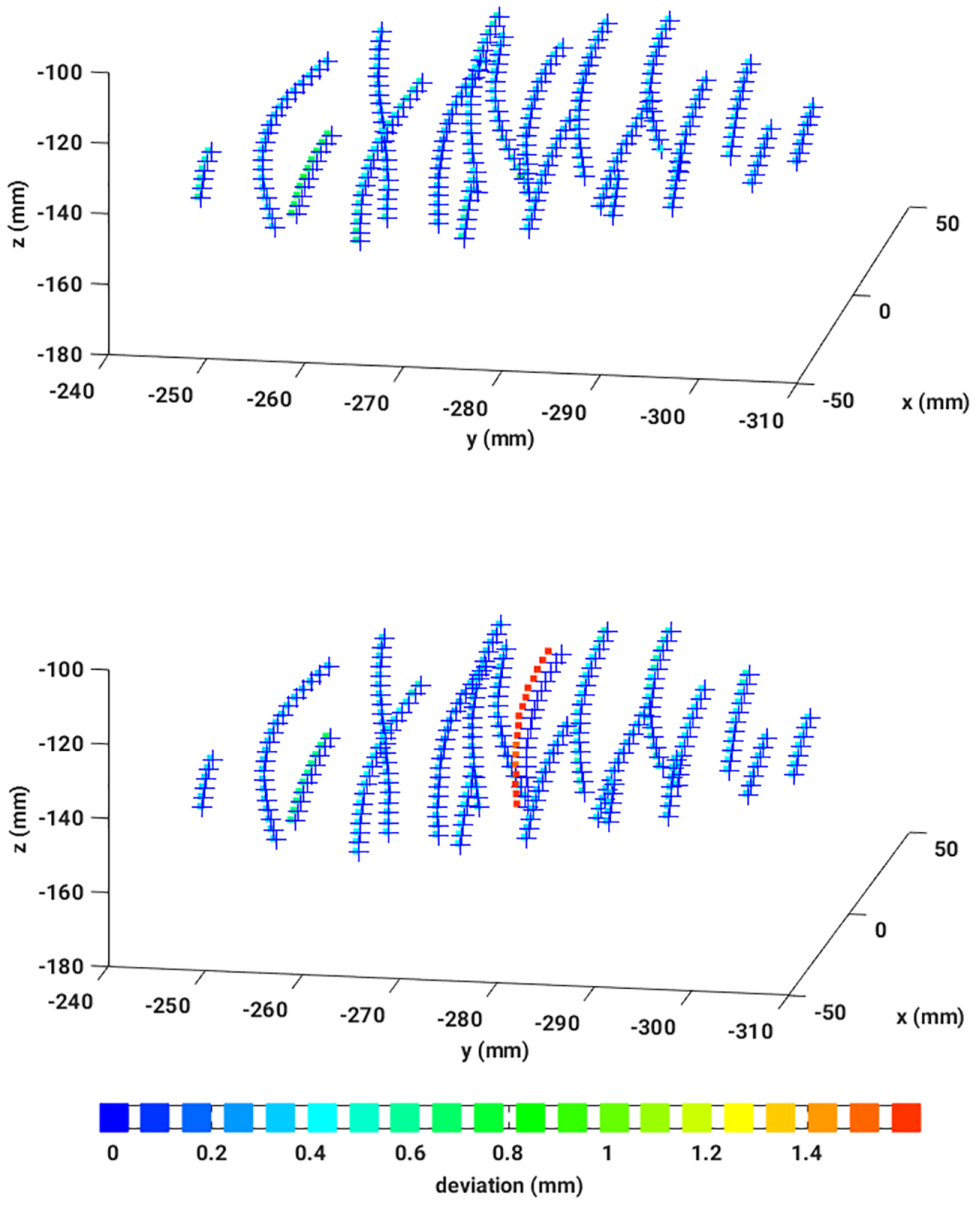
*Top:* Illustration of a perfect reconstruction of the catheter shapes from EMT measurements of a patient immediately after the CT: 3D dwell positions from the treatment plan (crosses), 3D reconstructed EMT dwell positions, colored according to their absolute deviations from the treatment plan. *Middle:* Illustration of a clear catheter shift revealed by EMT data reconstructed according to the signal processing chain proposed in this work. The data stem from the same patient after it has been moved to the EMT treatment room. Crosses signify 3D dwell positions from the treatment plan, while 3D reconstructed EMT dwell positions are drawn as squares colored according to the absolute deviations of the dwell positions from the treatment plan. *Bottom:* Colorbar.

**Fig 11:**
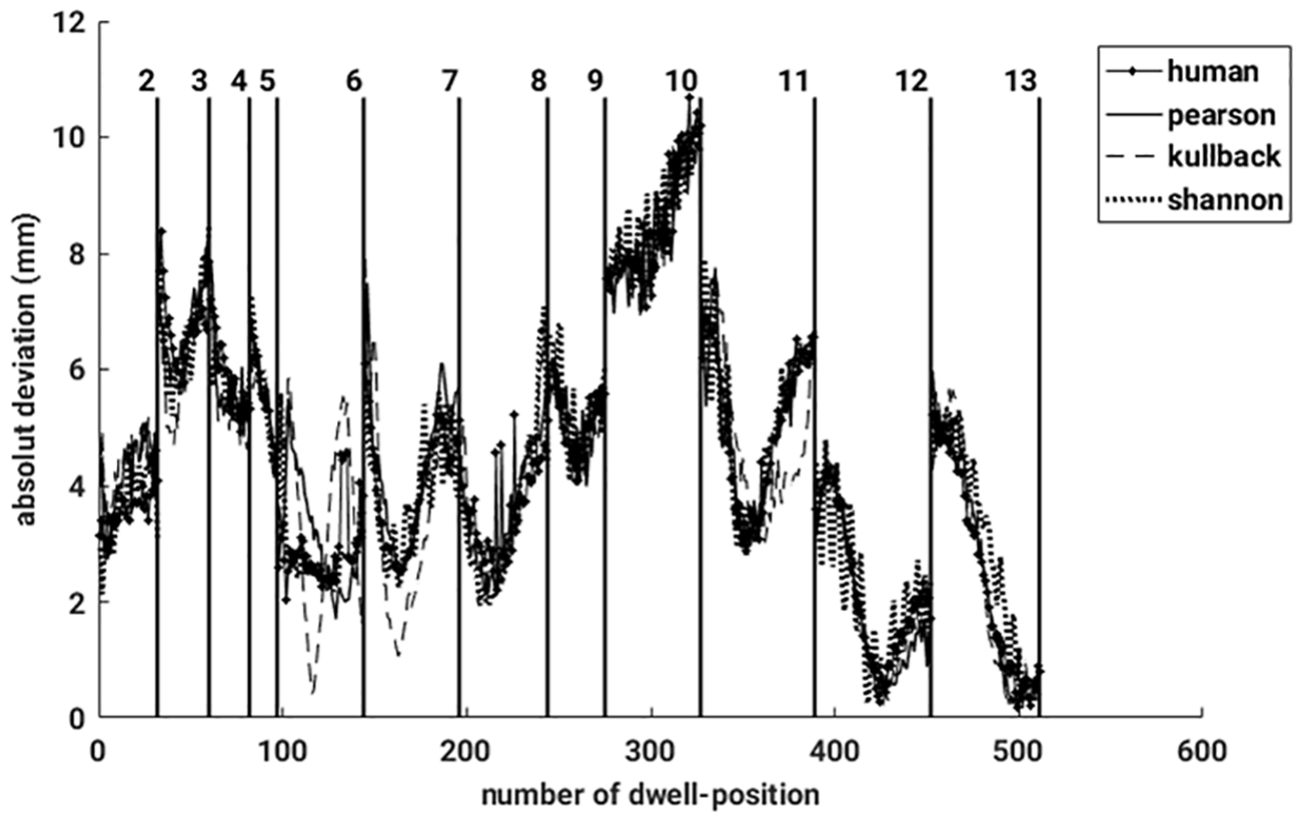
Absolute deviations for each pair of dwell positions (CT-EMT), are shown for four different reconstructed measurements. The black vertical lines mark the catheters.

**Fig 12:**
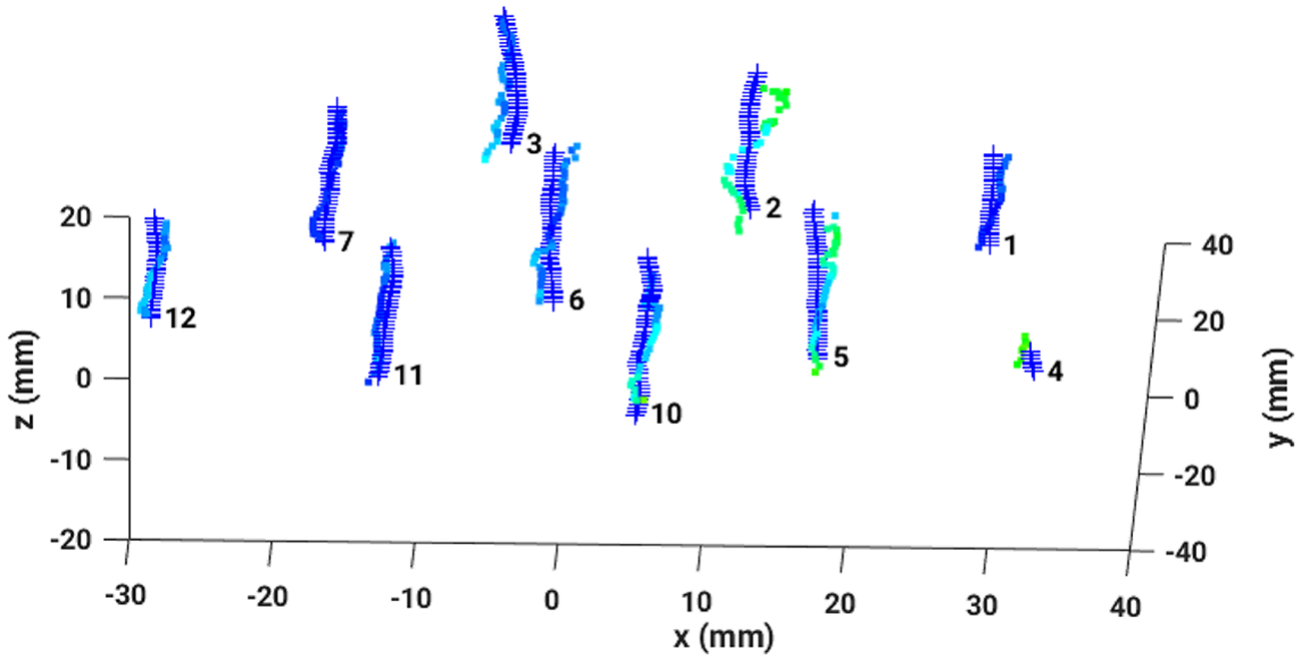
Three dimensional dwell positions from the treatment plan (crosses) and the reconstructed EMT measured dwell positions (colored according to the absolute deviation—See Fig 8 for the color bar). Reconstructed with the Jensen-Shannon divergence.

After reconstructing the data in four different ways, the dwell positions were compared with the dataset provided by the treatment plan. Applying the MDS-method, any mismatch of each pair of dwell positions could be quantified resulting in a sensitive detection of catheter displacements and bendings. A nice example is given in Fig 10 which shows EMT measurements of a female patient immediately after the X-ray CT has been taken (left graph) and hours later when the patient has been moved meanwhile to the EMT treatment room (right graph). The EMT measurements performed on the CT bench still do nor show any catheter deviations from the treatment plan. However, the second EMT measurement shows a clear catheter shift of one of the catheters, while for all others CT and EMT data coincide almost perfectly. The color coding indicates an average shift of 1.4 [*mm*] roughly. To summarize, in Fig 11, absolute dwell position deviations, resulting from four different similarity measures applied to remove breathing mode artifacts, are plotted against the number of dwell positions. Again, the vertical lines separate the different catheters. It is gratifying to see that all measured deviations are almost identical, hence results agree very well between the different methods. The data reconstructed by the human expert is very similar to the one deduced from the PCC. All in all, the observed differences between the three automatic methods are smaller than the measurement uncertainty. In case of the JSD, the results occasionally show strong fluctuations (for example see catheters number 12 or 13). A closer inspection of these data reveals that the breathing mode from this patient had a small amplitude, resulting in a low signal-to-noise ratio (SNR). Consequently, this breathing mode contamination could not be removed sufficiently well and introduced some noise in the reconstructed sensor signals. An example can be seen in Fig 12, where the same data as shown in Fig 8 was reconstructed with the aid of the JSD. In the catheters with number 3 or 2 breathing artifacts are still visible.

Next, an average over all deviations was determined. All the values are summarized in Table 2. Only small differences between the three different ways of reconstruction are observed. The mean deviations for the three phantom measurements are almost identical. Consequently, between the acquisition of the CT and the measurement on the male chest, a displacement of roughly 2.3 *mm* occurred. It is especially noteworthy that values resulting from the Jensen-Shannon divergence do not deviate much from the results of other similarity measures, although by employing the JSD, the breathing mode artifact could not always be removed completely. This is because the breathing mode amplitude is in most patients small compared to the dissimilarities which happen. Only for patient 1, the mean deviation is larger when determined using the JSD than when using other similarity measures.

**Table 2:** Collection of all mean deviations, measured in mm, for all datasets as obtained with four different similarity measures.

	pha 1	pha 2	pha 3	pat 1	pat 2	pat 3	pat 4
Human	2.44	2.31	2.01	7.30	4.31	10.74	12.32
PCC	2.51	2.26	1.91	7.17	4.41	10.73	11.98
KLD	2.56	2.33	1.96	7.21	4.33	10.60	12.03
JSD	2.45	2.27	2.01	7.57	4.41	10.80	11.87

In summary, the automatic removal of breathing artifacts worked very well for each dataset. Most importantly, it did not matter whether the patient was breathing while speaking or breathing calmly. The only disadvantage, data with breathing mode artifacts from speaking patients carry with them, is a loss of ‘stop’-information.

## Conclusion

High Dose Rate Brachytherapy (HDR-BT) encompasses the irradiation of a tumor bed after the cancerous tissue has been removed during a surgical intervention (Lumpectomy). During radiation therapy, a high activity radiation source will be positioned in close proximity to the tumor bed via catheters which are implanted surgically into the female breast. The spatial position of the radiation source inside these catheters is determined via an electromagnetic tracking (EMT) device. During radiation treatment, the source is moved inside the catheters while stopping at pre-defined dwell positions for predefined dwell times. The latter data are established, relative to the patient’s anatomy, from an initial X-ray computed tomography (CT) image by a medical physicist. During the radiation treatment sessions, which are scheduled during one week roughly, catheter displacements occur due to patient movements, breathing etc.. Assuring a proper radiation treatment in accord with the treatment plan, source positions need to be ascertained each time, a treatment session is scheduled. Such control measurements are performed using solenoid sensors instead of radiation sources whose movement inside the catheters follows the treatment plan and whose spatial positions are determined with an electromagnetic tracking (EMT) device. The current study develops an automatic analysis tool of such EMT data sets, recorded with a solenoid sensor, to either corroborate concordance of the source movement with the treatment plan or precisely quantify a spatial displacement that might have been occurred. The tool combines machine learning techniques such as

- a *particle filter* (PF) to precisely track the dwell positions of the solenoid sensor inside the catheters implanted in a female breast,
- a *singular spectrum analysis* (SSA) as a proper technique for removing high amplitude artifacts such as resulting from measurement device malfunctioning,
- an *ensemble empirical mode decomposition* (EEMD) to identify EMT signal components related to movement artifacts, especially breathing mode contributions even if the latter are highly erratic due to constantly speaking probands,
- linear and non-linear *similarity measures* such as Pearson correlation, Kullback—Leibler divergence and Jensen Shannon divergence to identify breathing mode contributions within EMT sensor signal recordings,
- a *multi-dimensional scaling* (MDS) to match positional measurements referring to different coordinate systems.

We convincingly demonstrate that the presented analysis tool allows to precisely detect and quantify any mismatch between the treatment plan and actual EMT measurements. We demonstrate that movement artifacts as well as technical signal distortions can be removed automatically and reliably, resulting in artifact-free reconstructed signals. This is a prerequisite for a highly accurate determination of any deviations of dwell positions from the treatment plan. Thus the tool offers a reliable control of the treatment plan and precisely quantifies any deviations from the latter before any treatment session is started.

## Appendix

### Appendix 1—Particle filter

The *Particle Filter* [38], [39] approximates the posterior probability density function *p*(**x**_0:*m*+1_|**z**_1:*m*_) by a *set of random samples*, called particles, with associated weights according to

$$\begin{array}{c} p\left(x_{0:m+1}|z_{1:m}\right)\approx \sum_{n=1}^{N}w_{m+1}^{n}\delta (x_{0:m+1}-x_{0:m+1}^{n}) \end{array}$$ (1)

where $w_{m+1}^{n}$ represents the weight of particle *n* having performed the particle trajectory $x_{0:m+1}^{n}$. Often only the marginal posterior density, called *filtering density*, is of interest and is given by

$$\begin{array}{c} p\left(x_{m+1}|z_{m}\right)\approx \sum_{n=1}^{N}w_{m+1}^{n}\delta (x_{m+1}-x_{m+1}^{n}) \end{array}$$ (2)

The application of a particle filter thus needs as input a *state evolution model* $x_{m+1}=f(x_{m},\epsilon_{m}^{\left(x\right)})$ and an observation model $z_{m+1}=h(x_{m+1},\epsilon_{m+1}^{\left(z\right)})$. Here $\epsilon_{m}^{\left(x\right)}$ and $\epsilon_{m+1}^{\left(z\right)}$ denote the state noise and the observation, i. e. measurement, noise, respectively. The model functions are given by (see [19] for details)

$$\begin{array}{c} f(l,t)=\text{RAL}+s+\frac{a}{2}+\sum_{i=1}^{7}(b_{i}\cos(\omega_{i}\cdot t)+c_{i}\sin(\omega_{i}\cdot t))+\epsilon^{\left(x\right)} \end{array}$$ (3)

$$h\left(l,x,y,z,t\right)=\theta +\epsilon^{\left(z\right)}\;\theta ={\left(l_{m},x_{m},y_{m},z_{m},t_{m}\right)}^{T}$$ (4)

Employing a sampling-importance-resampling scheme and sampling from the state transition probability density results in

- a particle update scheme, where the particles are drawn from the state transition probability density according to
  $$\begin{array}{c} p(x_{m+1}^{n}|z_{m})=w_{m}^{n}p(x_{m+1}^{n}|x_{m}^{n}) \end{array}$$ (5)
  $$\begin{array}{c} x^{n}∼p(x_{m+1}|x_{m}^{n}) \end{array}$$ (6)
- a weight update, where the weights are taken from the observation data likelihood according to
  $$\begin{array}{c} w_{m+1}^{n}=w_{m}^{n}p(z_{m+1}|x_{m+1}^{n}) \end{array}$$ (7)
  $$\begin{array}{c} w_{m+1}^{n}\propto p(z_{m+1}|x_{m+1}^{n}) \end{array}$$ (8)
  and the weight are finally normalized to sum to one.

Sampling from the data likelihood needs to be stabilized by resampling where particles with vanishing weights are replaced by copies of particles with finite weight. The new set of particles finally yields weights *N*^−1^ for each particle which results in the approximate posterior distribution

$$\begin{array}{c} p(x_{0:m},z_{1:m})=\sum_{n=1}^{N}\frac{c_{n}}{N}\delta (x_{0:m}-x_{0:m}^{n}) \end{array}$$ (9)

Further details are given in [19].

### Appendix 2—Singular spectrum analysis

Singular spectrum analysis (SSA) is a well-known signal analysis technique to solve such diverse problems as, for example, smoothing, detrending and extracting structures in short, noisy (chaotic) time series. It first appeared in [40], [41], became known more widespread through the work of [42], [43], but received public interest only after the seminal work of Ghil [28]. A thorough account of SSA was also provided by [29], while [31] discussed a linear invariant systems perspective of SSA. A decomposition of the original time series into a sum of orthogonal components is the aim of SSA. Thus second order correlations of the time series become decorrelated. Most of these components can be interpreted as trends, structureless white noise or oscillatory components. Furthermore, any parametric model of the considered time series has not to be known.

Let **x**(*t*) = (*x*(*t*_1_)⋯*x*(*t*_*T*_))^*T*^ ≡ **x**_*T*_ = (*x*_1_⋯*x*_*T*_)^*T*^ be a real-valued, zero mean time series with total length *T*. After selecting an embedding dimension *K* and a proper segment length *L* ≪ *T* such that *T* = *K* + *L* − 1, we have **x**_*k*_ = (*x*_*k*_, …, *x*_*k*+(*L*−1)_)^*T*^. Any analysis of such a time series with SSA requires two steps [44], [31]:

- a *decomposition step*, which encompasses embedding of the time series into *K* delayed coordinates combined with an eigendecomposition of a correlation matrix, and
- a *reconstruction step*, which encompasses anti-diagonal averaging and reverting the embedding.

#### Decomposition

- Embedding
  In general, time series represent uni-variate signals, while any decomposition technique requires multi-variate signals. Hence, a standard procedure in time series analysis is embedding the latter in its delayed coordinates. For that purpose, the uni-variate and zero mean time series segment **x**_*L*_ = (*x*_1_⋯*x*_*L*_)^*T*^ is transformed into a multi-variate set of delayed time series **x**_1_⋯**x**_*K*_ with column vectors $x_{k}={\left(x_{k}\cdots x_{k+L-1}\right)}^{T},k=1,\cdots ,K\in R^{L}$. The transformation, thus, is effected by an embedding of the original time series into delayed coordinates. Note that each time series has *L* entries, but the embedding space has dimension *K* < *L* ≪ *T*, and it is this latter space on which all the discussion to follow will concentrate.
  The result of this embedding step is an (*L* × *K*)—dimensional *trajectory matrix* given by [44]
  $$\begin{array}{c} X=[x_{1}\cdots x_{K}]=[\begin{array}{ccccc} x_{1} & x_{2} & x_{3} & \cdots & x_{K}\\ x_{2} & x_{3} & x_{4} & \cdots & x_{K+1}\\ x_{3} & x_{4} & x_{5} & \cdots & x_{K+2}\\ \vdots & \vdots & \vdots & & \vdots \\ \vdots & \vdots & \vdots & & \vdots \\ x_{L} & x_{L+1} & x_{L+2} & \cdots & x_{L+K-1} \end{array}] \end{array}$$ (10)
  where the time series segment of length *L* ≤ *T* and its delayed versions form the columns of this trajectory matrix. These column vectors **x**_*k*_ are sometimes called *L—lagged vectors*. Note that by shifting the time series components *x*_*l*+*k*_, *k* = 1, …, *K*, the elements of this trajectory matrix along the anti-diagonals are equal, consequently **X** represents a *Hankel matrix*. For an alternative representation, where the time series segment of length *L* and its *K* delayed versions form the rows of the corresponding trajectory matrix, the diagonal elements become equal, thus yielding a *Toeplitz matrix* instead [31]. Note also that generally the segment length is larger than the embedding dimension, i. e. *L* > *K* holds in most SSA applications—a setting that we will assume to apply throughout this study.
- Eigendecomposition
  Considering the matrix dot product **X**^*T*^ **X**, we obtain a *K* × *K*—dimensional, symmetric and real-valued correlation matrix **R** which possesses an eigendecomposition according to
  $$\begin{array}{c} (X^{T}X)v_{k}=Rv_{k}=\lambda_{k}v_{k} \end{array}$$
  where λ_1_ ≥ … ≥ λ_*K*_ denote the non-zero, ordered eigenvalues and **v**_*k*_ the corresponding eigenvectors. These eigenvectors (**v**_1_⋯**v**_*K*_) are orthogonal, i. e. $v_{k}^{T}v_{k^{\prime }}=0$ *for* *k* ≠ *k*′ and normalized to unity ||**v**_*k*_|| = 1, where ||.|| denotes the *L*_2_—norm. Note that this ordered set of eigenvalues {λ_*k*_, *k* = 1, …, *K*} represents the eigenspectrum of the trajectory matrix **X**.

Note that two possible representations of the matrix **X**^*T*^ **X** exist. One possible representation puts emphasis onto correlations along the time series segment with sample size *L*. We have

$$X^{T}X=\left[\begin{array}{c} {\left(x^{\left(L\right)}\right)}_{1}^{T}\\ \vdots \\ {\left(x^{\left(L\right)}\right)}_{K}^{T} \end{array}\right]\;\cdot \;\left[x_{1}^{\left(L\right)}\cdots x_{K}^{\left(L\right)}\right]\;=\;{\left[{\left(x_{k}^{\left(L\right)}\right)}^{T}x_{k^{\prime }}^{\left(L\right)}\right]}_{k,k^{\prime }=1}^{K}$$ (11)

In detail, this reads

$$\begin{array}{c} X^{T}X=[\begin{array}{cccccc} x_{1} & x_{2} & x_{3} & \cdots & \cdots & x_{L}\\ x_{2} & x_{3} & x_{4} & \cdots & \cdots & x_{L+1}\\ x_{3} & x_{4} & x_{5} & \cdots & \cdots & x_{L+2}\\ \vdots & \vdots & \vdots & & & \vdots \\ x_{K} & x_{K+1} & x_{K+2} & \cdots & \cdots & x_{L+K-1} \end{array}]\cdot [\begin{array}{ccccc} x_{1} & x_{2} & x_{3} & \cdots & x_{K}\\ x_{2} & x_{3} & x_{4} & \cdots & x_{K+1}\\ x_{3} & x_{4} & x_{5} & \cdots & x_{K+2}\\ \vdots & \vdots & \vdots & & \vdots \\ \vdots & \vdots & \vdots & & \vdots \\ x_{L} & x_{L+1} & x_{L+2} & \cdots & x_{L+K-1} \end{array}] \end{array}$$

Hence, the resulting matrix measures *correlations* along the *L*—dimensional time series. To see this consider the *auto-correlations* of the time series segment of length *L*, providing an estimate of the auto-correlation function *R*_*kk*′_ with a segment of size *L* < *T*, (*k*, *k*′ = 1, …, *K*)

$$\begin{array}{c} R_{kk^{\prime }}^{\left(L\right)}={\left(x_{k}^{\left(L\right)}\right)}^{T}x_{k^{\prime }}^{\left(L\right)}=\left(x_{k}^{\left(L\right)}\cdots \cdots x_{k+L-1}^{\left(L\right)}\right)(\begin{array}{c} x_{k^{\prime }}^{\left(L\right)}\\ \vdots \\ \vdots \\ x_{k^{\prime }+L-1}^{\left(L\right)} \end{array})=\sum_{l=1}^{L}x_{l-1+k}x_{l-1+k^{\prime }} \end{array}$$

Here the upper index (*L*) indicates the segment size.

An alternative representation of the matrix **X**^*T*^ **X**, formed with the trajectory matrix **X** given above, is the following

$$X^{T}X=\left[x_{1}^{\left(K\right)}\cdots x_{L}^{\left(K\right)}\right]\cdot \left[\begin{array}{c} {\left(x^{\left(K\right)}\right)}_{1}^{T}\\ \vdots \\ {\left(x^{\left(K\right)}\right)}_{L}^{T} \end{array}\right]\;=\;\overset{L}{\underset{l=1}{\sum }}\left[x_{l}^{\left(K\right)}{\left(x_{l}^{\left(K\right)}\right)}^{T}\right]$$ (12)

where the upper index (*K*) again denotes the segment size. Each segment now represents an element of the original time series and its *K* delayed versions. In detail, this representation reads

$$\begin{array}{ccc} X^{T}X & = & (\begin{array}{c} x_{1}\\ x_{2}\\ x_{3}\\ \vdots \\ x_{K} \end{array})\cdot (\begin{array}{ccccc} x_{1} & x_{2} & x_{3} & \cdots & x_{K} \end{array})+\cdots \\ & & +(\begin{array}{c} x_{L}\\ x_{L+1}\\ x_{L+2}\\ \vdots \\ x_{L+K-1} \end{array})\cdot (\begin{array}{ccccc} x_{L} & x_{L+1} & x_{L+2} & \cdots & x_{L+K-1} \end{array}) \end{array}$$

Thus the matrix **X**^*T*^ **X** is written as a sum of rank 1 outer product matrices generated by data vectors living in the embedding space with dimension *K*. As we have seen above, it is in this space where we have to consider the eigenvalue decomposition of the matrix **X**^*T*^ **X**.

Having obtained the eigenvectors **V**, we can consider the projections of the data onto them yielding

$$\begin{array}{c} z_{k}=Xv_{k} \end{array}$$

#### Reconstruction

Next we can reconstruct the data by forming the outer product component matrices

$$\begin{array}{c} X_{k}=z_{k}v_{k}^{T} \end{array}$$

- Dimension reduction
  If dimension reduction is intended, the best approximation to the trajectory matrix **X** is provided by the matrix $\sum_{k=1}^{R}X_{k}$ with *R* < *K*. The minimum is represented by ||**X** − **X**^(*R*)^||, where $\left|\right|X_{k}{\left|\right|}^{2}=X_{k}^{T}X_{k}=\lambda_{k}$ and ${\left||X|\right|}^{2}=\sum_{k=1}^{K}\lambda_{k}$ for *k* = 1, …, *K*.
  Often the number of relevant eigenvalues λ_1_, …, λ_*R*_ and related component matrices **X**_*k*_ can be estimated from a scree—plot. There, a knee or bend in a graph λ_*k*_ *vs* *k* indicates the number of intrinsic harmonics versus noise or aperiodic signal components [45]. Signal reconstruction can then be pursued neglecting noise contributions and even trends and other aperiodic signal components. The eigenvalue ratio $\sum_{k=1}^{R}\lambda_{k}/\sum_{k=1}^{K}\lambda_{k}$ quantifies the approximation of the trajectory matrix by the matrices of rank *R*.
- Anti-diagonal averaging
  Note that after neglecting non-harmonic signal components, the resulting matrix **X**^(*R*)^ does not correspond to a trajectory matrix anymore. To reconstitute a trajectory matrix, anti-diagonal averaging is invoked. This can be achieved simply by averaging over all elements along every anti-diagonal of the component matrices ${\left[X_{k}\right]}_{k=1}^{R}$. Replacing each element of every anti-diagonal by just its average renders the reconstructed matrix ${\hat{X}}^{\left(R\right)}$ a Hankel matrix again.
  After having reconstituted a Hankel matrix, an approximation to the original time series can be obtained by concatenating from the reconstructed trajectory matrix its first row and its last column.
  SSA will be used in this study to remove large amplitude artifacts from sensor signals, both fiducial and solenoid, measured with EMT. Typically, such artifacts dominate the signal decomposition and correspond to the principal mode related with the largest eigenvalue.

### Appendix 3—Ensemble empirical mode decomposition and Fourier spectrum

#### Ensemble empirical mode decomposition

Natural time series often happen to be non-stationary and non-linear. Exploratory data analysis techniques like principal (PCA) or independent component analysis (ICA), non-negative matrix (NMF) or tensor factorization (NTF) have limitations to analyze such data as they assume at least wide-sense stationarity. However, in 1998 N. E. Huang et al. [33] invented an empirical mode decomposition (EMD) which represents any time series as a superposition of components with well defined instantaneous frequencies. They adaptively and locally decompose any non-stationary signal in a sum of intrinsic modes (IMFs), which represent zero-mean, amplitude- and frequency-modulated components, plus a non-oscillating trend according to

$$\begin{array}{c} r_{\epsilon }^{\left(p,r\right)}\left(t\right)=r^{\left(p,r\right)}\left(t\right)+\epsilon_{n}\left(t\right)=\sum_{j=1}^{J}c_{n}^{\left(j\right)}\left(t\right),\;n\in N \end{array}$$ (13)

$$\begin{array}{c} c^{\left(j\right)}\left(t\right)=Re\{a_{j}\left(t\right)\exp(i\int_{-\infty }^{t}\omega_{j}\left(t^{\prime }\right)dt^{\prime })\} \end{array}$$ (14)

Here *r*^(*p*, *r*)^(*t*) represents one component of the tracked sensor signal, *ϵ*(*t*) denotes the random noise deliberately added to the signal at any iteration, *c*^(*j*)^(*t*) represents the ensemble-averaged *j*-th intrinsic component, *c*^(*J*)^(*t*) the non-oscillating trend, *a*_*j*_(*t*) the time-varying amplitude and *ω*_*j*_(*t*) the instantaneous frequency. Note that EMD is not based on any *a priori* defined basis system and that it obeys the perfect reconstruction property. Thus EMD lacks the scaling and permutation indeterminacy familiar from exploratory matrix decomposition techniques. An informative illustration of the main steps of an EEMD algorithm can be found in [46], and [47] provides a toolbox for a convenient application of various EEMD variants to time series analysis problems.

#### Fourier Transformation (FT) and Fourier spectrum (FS)

The spatial position of the EMT sensor is measured while it is moved according to a treatment plan. Consequently, the sensor stopped at all dwell positions and remained there in accord with the dwell time definitions. The latter lasted from 0.1 *s* to 5 *s*. These dwell positions and dwell times form a very important information which cannot be lost during the reconstruction of the catheter track. To ensure this, a Fast Fourier Transformation (FFT) is applied to each IMF

$$\begin{array}{c} c^{\left(j\right)}\left(n\cdot T_{s}\right)=(c^{\left(j\right)}\left(0\right),\ldots ,c^{\left(j\right)}\left(\left(T-1\right)\cdot T_{s}\right)) \end{array}$$

where $T_{s}^{-1}$ *Hz* = 40 *Hz* denotes the sampling rate and *n* = 0, …, *T* − 1 denotes the number of samples in each segment. This yields

$$\begin{array}{c} C^{\left(j\right)}\left(r\cdot \omega_{s}\right)=\sum_{n=0}^{T-1}c^{\left(j\right)}\left(n\right)\exp(-ir\omega_{s}\frac{nT_{s}}{T}) \end{array}$$

where *ω*_*s*_ = 2*π*/*T*_*s*_ is the sampling frequency which, according to the Nyquist theorem has to obey the relation *ω*_*s*_ ≥ 2*ω*_*max*_. The absolute values |*C*^(*j*)^(*r*)| of the Fourier spectral amplitudes of the high and low frequency modes extracted from the EMT sensor signal (see Fig 5) are illustrated in Fig A in S1 File. The Fourier spectra of the intrinsic modes *c*^(1)^ and *c*^(2)^ resemble broadband signals with a peak at rather high frequencies, whereas the Fourier spectra of the low frequency IMFs resemble narrow-band signals whose bandwidth shrinks with decreasing dominant mode frequency. Note that the stop-and-go mode of movement of the sensor inside a catheter results in abrupt displacements which entails high frequency modes in each frequency resolved decomposition. Consequently, the highest frequency IMFs contain information about the dwell positions. Hence, they should not be neglected during the reconstruction of the signal. Digging out, which IMF provides information about the dwell times and dwell positions, the area under a Fourier spectrum is calculated in the following way:

$$\begin{array}{c} A(|C_{k}^{\left(j\right)}|)=\sum_{r=0}^{T-1}\frac{|C^{\left(j\right)}\left(r\cdot \omega_{s}\right)|}{T\cdot T_{s}} \end{array}$$ (15)

If the area is larger than *A* > *A*_*th*_ = 0.04, where the threshold *A*_*th*_ has been determined empirically, then IMF *c*^(*j*)^ is included in the reconstruction of the sensor signal. Fig 7 clearly demonstrates that information about dwell positions is contained in the resulting signal. In Fig B in S1 File, two reconstructed catheters and the measured EMT-signal (blue stars) can be seen. The reconstructed trace illustrated with black stars allows the stop positions to be identified, whereas the reconstructed trace symbolized by the green dots lacks information about the *stop* positions, and a continuous line through the measurements is seen.

### Appendix 4—Similarity measures

#### Linear correlations

An often used similarity measure between two data sets considers a pointwise, linear correlation between the variables. The underlying statistic of the stochastic variables follow a normal distribution. Though not always encountered in practice, it is often a reasonable first order approximation to estimate the similarity between the two stochastic processes.

Pearson correlation: One of the most popular measures of correlation between variables is the *Pearson correlation coefficient*. It was first described by Karl Pearson in [48]. Let **x** = (*x*_1_⋯*x*_*L*_)^*T*^ and **y** = (*y*_1_⋯*y*_*L*_)^*T*^ be two time series segments with size *L* ≤ *T*, represented as vectors in an *L*-dimensional space. The definition of the Pearson correlation coefficient *c* is as follows:

$$\begin{array}{c} PCC\left(x,y\right)=\frac{L\sum_{l=1}^{L}x_{l}y_{l}-\left(\sum_{l=1}^{L}x_{l}\right)\left(\sum_{l=1}^{L}y_{l}\right)}{\sqrt{L\sum_{l=1}^{L}x_{l}^{2}-{\left(\sum_{l=1}^{L}x_{l}\right)}^{2}}\sqrt{L\sum_{l=1}^{L}y_{l}^{2}-{\left(\sum_{l=1}^{L}y_{l}\right)}^{2}}} \end{array}$$ (16)

The Pearson correlation coefficient can vary between −1 and 1 [49], [50] and helps to identify the latent intrinsic mode of the sensor signals most similar to the breathing mode artifact.

#### Non-linear correlations

If linear correlations do not apply, similarity between two stochastic processes should be based on metrics defined by distributions of the variables in the data sets. The basis of such metrics is laied by the information-theoretic entropy, also known as Shannon entropy [51], [52]. A related measure based on it is the mutual information and several divergences [53], which are shortly summarized next.

- Shannon entropy and Mutual information
  In information theory, entropy measures the average surprise that comes along with an event *x*_*l*_ with occurrence probability *p*(*x*_*l*_). The expected information, the event carries with it, is known as *Shannon entropy* and is given by
  $$\begin{array}{c} H\left(X\right)=H\left(p\left(x_{l}\right)\right)=\sum_{l}p\left(x_{l}\right)\;\log(\frac{1}{p(x_{l})})=-\sum_{l}p\left(x_{l}\right)\log\left(p\left(x_{l}\right)\right) \end{array}$$
  where *X* represents a stochastic variable whose realizations are denoted by **x**^*T*^ = (*x*_1_, *x*_2_, …, *x*_*L*_). Information entropy, thus, can be interpreted as a measure of uncertainty, i. e. the amount of information an event carries with when taking place, and the dispersion of the probabilities with which the events take place.
  Related to Shannon entropy is *mutual information* (MI) between two signals, say *X* ∼ *p*(**x**) and *Y* ∼ *p*(**y**), and is defined as
  $$\begin{array}{c} I(Y,X)=I(p(y),p(x))=H(Y)-H(Y|X)=H(X)-H(X|Y)=I(X,Y) \end{array}$$
  where *X* and *Y* denote multi-variate stochastic variables with related joint probability densities *p*(**x**) and *q*(**y**), respectively. For example, *X* denotes an intrinsic mode of an EMT sensor signal and *Y* denotes an intrinsic mode of a fiducial sensor signal. MI measures the amount of information, an EMT sensor mode carries about a fiducial sensor mode [54, 55]. In multi-channel biomedical systems, MI is most frequently used to measure the amount of independence between both modes. Hence, the most convenient property of mutual information is its disappearance if and only if *X* and *Y* are statistically independent. Or, vice versa and relevant for our considerations, the larger *I*(*X*, *Y*) is, the more similar both signals are, i. e. the less uncertain we are about one signal knowing the other. Contrary to Pearson correlation (PC), Shannon entropy and MI both rely on distributions rather than pointwise comparisons, where non-linear correlations are contained in the distributions. Note that in case of signals having Gaussian distributions, both similarity measures, i. e. PC and MI, become equivalent. However, as MI has a lower bound only, corresponding to statistical independence of both signals, it is less well suited to directly measure similarity between signals.
- Kullback—Leibler divergence
  The *Kullback—Leibler divergence* (KLD) [56], also called relative Entropy, is a non-symmetric measure of similarity between two distributions *P* and *Q*
  $$\begin{array}{c} D_{KL}\left(X∥Y\right)\equiv D_{KL}\left(p\left(x\right)∥q\left(y\right)\right)=\sum_{l=1}^{L}p\left({\hat{x}}_{l}\right)\ln(\frac{p({\hat{x}}_{l})}{q({\hat{y}}_{l})}) \end{array}$$
  where the stochastic variables need to be normalized according to
  $$\begin{array}{c} {\hat{y}}_{l}=\frac{y_{l}}{\sum_{l=1}^{L}y_{l}}\;\text{and}\;{\hat{x}}_{l}=\frac{x_{l}}{\sum_{l=1}^{L}x_{l}} \end{array}$$
  Occasionally it is said to measure the distance between two distributions but this is unfortunate as KLD does not fulfill the triangle inequality. However, it can be understood as the loss of information if *p*(**x**) is modeled by means of *q*(**y**). If both are equal, there is no loss and the KLD becomes zero. Thus it offers a very convenient measure of similarity, though not really corresponding to a valid distance metric, and we have
  $$\begin{array}{c} D_{KL}\left(P∥Q\right)\geq 0\;\text{and}\;D_{KL}\left(P∥Q\right)=0\;\text{iff}\;p\left(x\right)=q\left(y\right) \end{array}$$ (17)
  Alternatively, the KLD tells one the amount of information obtained per observation of *X* that allows one to discriminate between the two distributions *p*(**x**) and *q*(**y**). In this study, *p*(**x**) might represent an intrinsic mode from the EMT sensor signal, and *q*(**y**) might represent an intrinsic mode from the fiducial sensor signal. The goal is to identify the intrinsic mode from the EMT sensor signal which most closely resembles the breathing mode from the fiducial sensor signal. Hence, in practice, *p*(**x**) and *q*(**y**) are derived from observations and sample counting. That is, *p*(**x**) and *q*(**y**) are probability distributions derived from frequency distributions. The derivation of a probability distribution from an observed frequency distribution is called smoothing. If one of the binned distributions contains zeros as entries, one can replace them by a small quantity *ϵ* ≤ 10^−3^. Related with this, an important assumption is that *p*(*x*_*l*_) = 0 ⇔ *q*(*y*_*l*_) = 0 which furthermore implies that in this case $p\left(x_{l}\right)\ln(\frac{p(x_{l})}{q(y_{l})})=0$. A general convention is that 0ln(0/*q*(*y*)) = 0 for any *q*(*y*), and *p*(*x*)ln(*p*(*x*)/0 = ∞ if *p*(*x*)>0.
  Considering the relation of relative entropy *D*_*KL*_ to mutual information *I*(*X*, *Y*), we have for two stochastic variables *X* and *Y*
  $$\begin{array}{ccc} I(p(x),p(y)) & = & D_{KL}(p(x,y)∥p(x)p(y))\\ & = & \sum_{l,l^{\prime }=1}^{L}p\left(x_{l},y_{l^{\prime }}\right)\ln(\frac{p(x_{l},y_{l^{\prime }})}{p\left(x_{l}\right)p\left(y_{l^{\prime }}\right)}) \end{array}$$ (18)
- Jensen—Shannon divergence
  The *JensenShannon divergence* (JSD) [57], [58] is another similarity measure between two probability distributions. It is based on the *KullbackLeibler divergence* (KLD) but it is symmetric and its value is always finite. Indeed, the JSD is a symmetrized and smoothed version of the KLD. Furthermore, the square root of the JensenShannon divergence is a metric often referred to as *Jensen-Shannon distance* $d_{JS}=\sqrt{JSD}$.
  Given two realizations *p*(*x*), *q*(*y*) of discrete probability distributions *P*, *Q*, the *JSD*(*P* ∥ *Q*) is defined as
  $$\begin{array}{c} JSD(P∥Q)=\frac{1}{2}D_{KL}(P∥\frac{P+Q}{2})+\frac{1}{2}D_{KL}(Q∥\frac{P+Q}{2}) \end{array}$$ (19)
  $$\begin{array}{c} =H(\sum_{n=1}^{N}w_{n}P_{n})-\sum_{n=1}^{N}w_{n}H(P_{n}) \end{array}$$ (20)
  where, for the second equality, *N* = 2, *P* ≡ *P*_1_, *Q* ≡ *P*_2_ is used and *w*_1_ = *w*_2_ = 1/2. This latter relation, obviously, can be trivially generalized to more than two distributions. This definition shows that the JSD is a symmetrized version of two KLDs measuring the similarity of each of the two considered distributions with their corresponding mixture distribution. The JSD obeys the following bounds:
  $$\begin{array}{ccc} 0\leq JSD(P,Q)\leq \ln(2) & \text{if} & \;\log_{e}\;\;\text{is}\;\text{used}\\ 0\leq JSD(P,Q)\leq 1\; & \text{if} & \;\log_{2}\;\;\text{is}\;\text{used} \end{array}$$
  The Jensen—Shannon divergence is intimately related to mutual information. To see that consider a superposition of the two distributions *X* = (*P* + *Q*)/2, where *p*(*x*) represents the distribution related with an intrinsic mode of the EMT sensor signal and *q*(*y*) represents a distribution related with an intrinsic mode of the fiducial sensor signal. Consider next an indicator variable *Z*|*z* ∈ (0, 1). Let the indicator variable *Z* be used to switch between the two distributions, i. e. choose *p*(*x*) if *z* = 0 and *q*(*x*) if *z* = 1. Then we obtain for the mutual information *I*(*X*, *Z*) the following relation
  $$\begin{array}{c} 0\leq I(X,Z)=H(X)-H(X|Z)=JSD(P,Q)\leq 1 \end{array}$$ (21)
  Hence, if both distributions become identical, *P* = *Q*, then the conditional information entropy *H*(*X*|*Z*) becomes independent of the indicator variable *Z* and the mutual information *I*(*X*, *Z*) becomes zero. This implies that also the JSD and its related JS distance become zero.

### Appendix 5—Multi-dimensional scaling

In HDR-BT dwell positions of a radiation source inside catheters implanted into a female breast are defined by a treatment plan which is deduced from an initial X-ray CT image. As a radiation treatment is repeated during a couple of subsequent days, dwell positions need to be checked by a EMT measurement inserting a solenoid sensor into the catheters before the radiation treatment. The spatial coordinates of the sensor at the various dwell positions in the magnetic field of a field generator are recorded. However, such coordinates refer to a different coordinate system in each session. As we proposed recently, such difficulties can be alleviated by recourse to multi-dimensional scaling techniques which only rely on distances (see [15] for details).

Denote by $X_{p,r}^{EMT}$ the set of dwell position coordinates registered in catheter *p* during session *r* and by $X_{0}^{CT}$ the related set of coordinates of the original treatment plan. Define a matrix of dissimilarities according to

$$\begin{array}{ccc} {\left(D\right)}_{mm^{\prime }}=-\frac{1}{2}d^{2}\left(x_{m},x_{m^{\prime }}\right) & = & -\frac{1}{2}{\left(x_{m}-x_{m^{\prime }}\right)}^{T}\left(x_{m}-x_{m^{\prime }}\right)\\ & = & -\frac{1}{2}\left(k_{mm}+k_{m^{\prime }m^{\prime }}-2k_{mm^{\prime }}\right) \end{array}$$ (22)

Double-centering matrix **D**^(*c*)^ renders it identical to a centered kernel matrix **K**^(*c*)^, whose eigendecomposition provides an eigenvector system **V**, which allows for a spectral representation of the dwell positions in an axis system spanned by the eigenvectors of the related centered covariance matrix according to $\hat{X}=\Lambda^{1/2}V^{T}$. The technique allows to precisely quantify any deviations of a dwell position of the sensor in any of the catheters during the session under consideration.

## Supporting information

S1 File Fig A, Fourier spectra. Fig B, Sensor EMT signal and stop positions.
(PDF)
