# Supplementary material for: A tool to automatically analyze electromagnetic tracking data from high dose rate brachytherapy of breast cancer patients
Source: PLoS One. 2017 Sep 21;12(9):e0183608. doi: 10.1371/journal.pone.0183608 (PMC5608198; doi:10.1371/journal.pone.0183608)
Supplement: S1 File — Fig A, Fourier spectra. Fig B, Sensor EMT signal and stop positions. (PDF) [file pone.0183608.s001.pdf]

A Tool to Automatically Analyze Electromagnetic Tracking Data from High Dose Rate Brachytherapy of Breast Cancer Patients
Supplementary Material

Th. I. Götz[1,2,4,*], G. Lahmer[2], V. Strnad[2], Ch. Bert[2], B. Hensel[4], A. M. Tomé[3] and E. W. Lang[1],
1 CIML, Biophysics, University of Regensburg, 93040 Regensburg, Germany
2 Department of Radiation Oncology, Universitätsklinikum Erlangen, Friedrich-Alexander-Universität Erlangen-Nürnberg, 91054 Erlangen, Germany
3 IEETA, DETI, Universidade de Aveiro, 3810-193 Aveiro, Portugal
4 Center for Medical Physics and Engineering, University of Erlangen-Nuremberg, 91052 Erlangen, Germany
∗ E-mail: theresa.goetz@biologie.uni-regensburg.de

Supporting information

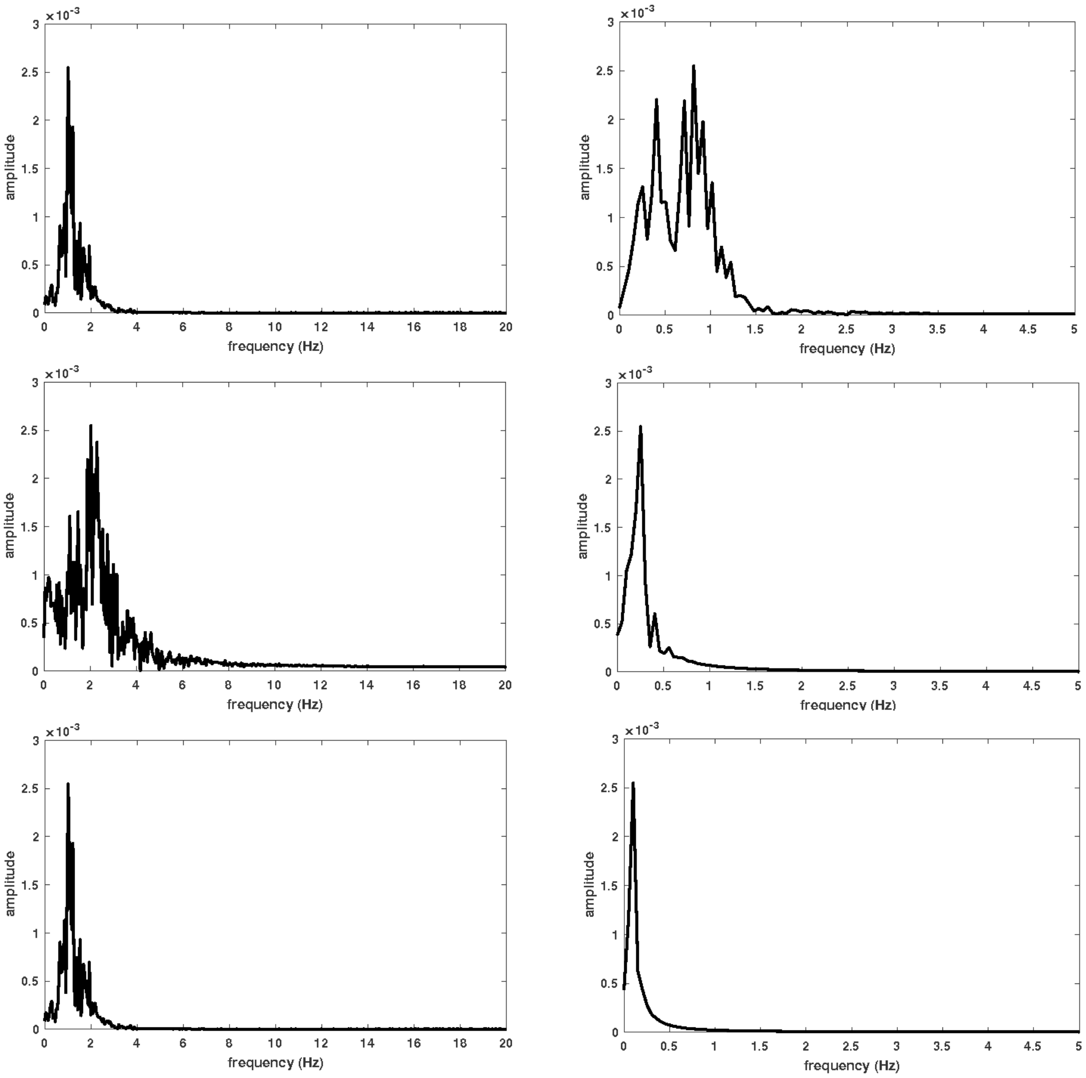

Figure 1. Fourier spectra. Absolute values of the Fourier spectra of the IMFs c_1 (top left) to c_6 (bottom right) are shown. On the x-axis the frequency and on the y-axis the amplitude is plotted.

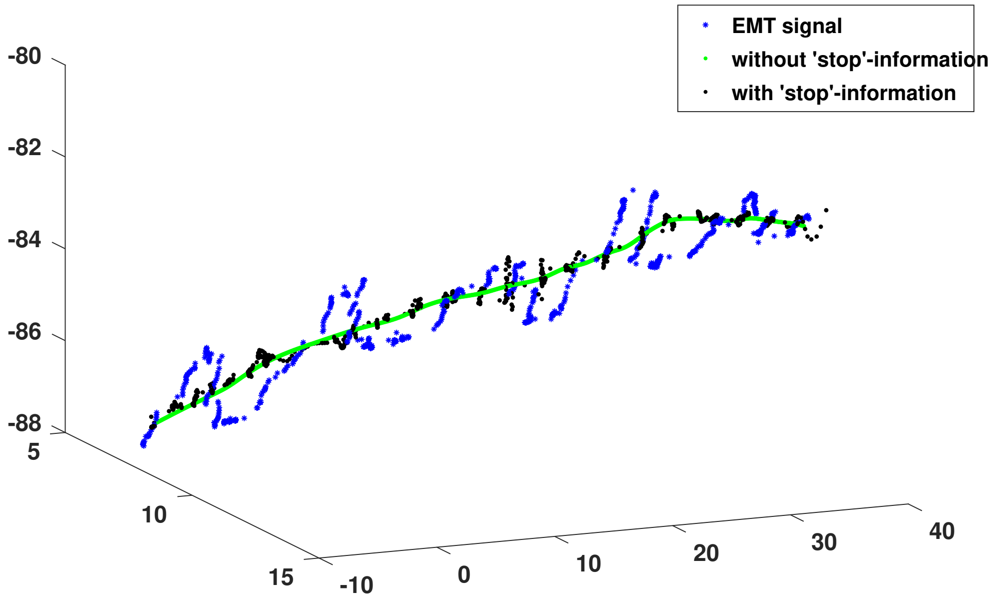

Figure 2. Sensor EMT signal and stop positions. EMT measured signal from one catheter (blue), the signal after reconstruction with 'stop'-information (black) and the reconstructed signal without 'stop'-information (green).
